# A Cluster Randomised Controlled Effectiveness Trial Evaluating Perinatal Home Visiting among South African Mothers/Infants

**DOI:** 10.1371/journal.pone.0105934

**Published:** 2014-10-23

**Authors:** Mary Jane Rotheram-Borus, Mark Tomlinson, Ingrid M. le Roux, Jessica M. Harwood, Scott Comulada, Mary J. O'Connor, Robert E. Weiss, Carol M. Worthman

**Affiliations:** 1 Department of Psychiatry and Biobehavioral Medicine, Semel Institute, University of California Los Angeles, Los Angeles, California, United States of America; 2 Department of Psychology, Stellenbosch University, Stellenbosch, South Africa; 3 Philani Maternal, Child Health and Nutrition Programme, Elonwabeni, Cape Town, South Africa; 4 Department of Anthropology, Emory University, Atlanta, Georgia, United States of America; 5 Department of Biostatistics, UCLA Fielding School of Public Health, University of California Los Angeles, Los Angeles, California, United States of America; Columbia University, United States of America

## Abstract

**Background:**

Interventions are needed to reduce poor perinatal health. We trained community health workers (CHWs) as home visitors to address maternal/infant risks.

**Methods:**

In a cluster randomised controlled trial in Cape Town townships, neighbourhoods were randomised within matched pairs to 1) the control, healthcare at clinics (n = 12 neighbourhoods; n = 594 women), or 2) a home visiting intervention by CBW trained in cognitive-behavioural strategies to address health risks (by the Philani Maternal, Child Health and Nutrition Programme), in addition to clinic care (n = 12 neighbourhoods; n = 644 women). Participants were assessed during pregnancy (2% refusal) and 92% were reassessed at two weeks post-birth, 88% at six months and 84% at 18 months later. We analysed 32 measures of maternal/infant well-being over the 18 month follow-up period using longitudinal random effects regressions. A binomial test for correlated outcomes evaluated overall effectiveness over time. The 18 month post-birth assessment outcomes also were examined alone and as a function of the number of home visits received.

**Results:**

Benefits were found on 7 of 32 measures of outcomes, resulting in significant overall benefits for the intervention compared to the control when using the binomial test (p = 0.008); nevertheless, no effects were observed when only the 18 month outcomes were analyzed. Benefits on individual outcomes were related to the number of home visits received. Among women living with HIV, intervention mothers were more likely to implement the PMTCT regimens, use condoms during all sexual episodes (OR = 1.25; p = 0.014), have infants with healthy weight-for-age measurements (OR = 1.42; p = 0.045), height-for-age measurements (OR = 1.13, p<0.001), breastfeed exclusively for six months (OR = 3.59; p<0.001), and breastfeed longer (OR = 3.08; p<0.001). Number of visits was positively associated with infant birth weight ≥2500 grams (OR = 1.07; p = 0.012), healthy head-circumference-for-age measurements at 6 months (OR = 1.09, p = 0.017), and improved cognitive development at 18 months (OR = 1.02, p = 0.048).

**Conclusions:**

Home visits to neighbourhood mothers by CHWs may be a feasible strategy for enhancing maternal/child outcomes. However, visits likely must extend over several years for persistent benefits.

**Trial Registration:**

ClinicalTrials.gov NCT00996528

## Introduction

Pregnancy and early childhood is a critical developmental period that creates lifelong advantages or costs for children living in poverty, including in South Africa [1]. While the specific sources of risk vary by country, the intervention approaches that demonstrably improve outcomes for pregnant women and infants share common characteristics. Efficacious programs establish a supportive relationship, provide knowledge, but help women apply the new knowledge to their daily life, and problem solve challenges to healthy daily routines [2]. Women in South Africa face challenges about preventing HIV transmission, stopping alcohol use during pregnancy, preventing and managing pre-term births and low birth weight (LBW) infants, and sustaining infant growth [3]–[5].

The most robust intervention to date for low income, high risk mothers has been nurse home visiting over the first two years of life by Olds and colleagues in the US [6], which produced benefits lasting until early adulthood [7]–[9]. When community health workers (CHWs) visit at-risk mothers in the US [10], the outcomes appear to be less robust, compared to nurses. Low and middle-income countries (LMIC) such as South Africa, however, cannot afford nurses, and will not be able to train the personnel necessary to mount such support until at least 2050 [11], [12]. CHWs have been employed to bridge this gap in LMIC; however, their efforts are almost always focused on improving a single outcome. For example, there have been several perinatal intervention trials aimed at reducing maternal depression; yet the challenges are far broader than mothers' depression [13], [14].

The effects of poverty on children are pervasive and long-lasting [1]. Mothers in LMIC frequently lack the resources necessary for maintaining consistent and high quality nutrition, shelter, and health care; the relationships to buffer the stresses of childbearing and rearing; and the ability to create opportunities for their children to optimize their health and well-being. Given the range of challenges, especially in countries affected by epidemics of HIV, alcohol abuse, and malnutrition, CHW who aim to improve health outcomes must be trained as generalists who can support mothers to address multiple health challenges [15]. This study evaluates the effectiveness of CHW trained as generalists to improve maternal and child outcomes over the first 18 months of life.

We implemented a cluster randomised controlled trial in Cape Town townships to examine maternal and child outcomes when trained CHWs implement home visits to all women in a neighbourhood. Home visitors were selected from community role models and trained in basic strategies to change thoughts, feelings, and behaviours, and targeted the specific health challenges of South Africa [16], [17]. At the time of recruitment, 66% of the women in this study faced at least one health risk of having HIV, a history of alcohol abuse, depression or a previous low birth weight baby [15]. All pregnant women in a neighbourhood were visited, not only those at risk, to avoid contributing to household stigma, particularly associated with HIV.

Consistent with our trial design [16], we evaluated maternal and infant outcomes over 18 months using a primary evaluation strategy: the binomial test for correlated outcomes, based on 32 measures regarding HIV-related prevention, child health, maternal healthcare adherence, depression, and social support. Evaluations of the home visits' impact over the first six months of life have found benefits in multiple areas [17]. To examine if the early gains are sustained to 18 months, we examined the outcomes at the 18 month assessment alone, as well as the relationship between the number of home visits received and the outcomes.

## Methods

### Ethics statement

The Institutional Review Boards of University of California Los Angeles (UCLA), Stellenbosch University, and Emory University approved the study, whose methods have previously been published [16]. We received written informed consent from all study participants. Three independent teams conducted the assessment (Stellenbosch), intervention (Philani), and data analyses (UCLA). This cluster randomised control trial is registered with ClinicalTrials.gov (NCT00996528). The protocol for this trial and supporting CONSORT Checklist are available as supporting information; please see Protocol S1 and Checklist S1.

### Participants

#### Neighbourhood matching, randomisation, power, and recruitment

Aerial maps, observations, and street-intercept surveys of residents were conducted in order to match township neighbourhoods [16] outside Cape Town, South Africa on the types of housing (formal/informal), presence of electricity, running water, type of sanitation, the number of households and density, counts of alcohol bars (shebeens), child care resources, distance to clinics, length of residence, and original homeland area. UCLA randomised 26 neighbourhoods within matched pairs to either the intervention or the control arm using simple randomisation. One matched pair was eliminated after six months of recruitment due to low numbers of pregnant women (n = 13 combining both neighbourhoods, compared to n = 38–44 on average), leaving 24 study neighbourhoods [16], [17]. Because we were training CHW as generalists, we identified an analytic strategy that included multiple indices as the primary outcome, considering the base rate of each composite measure in each measure. Sample size calculations were conducted to determine the minimum number of pregnant women that would need to be recruited per clinic to achieve 80% power to detect a standardized effect size of 0.40 between women from the 12 intervention and 12 control neighborhoods on one overall summary measure, considering the anticipated base rate on each measure included in the index.

Pregnant women were identified by recruiters conducting house-to-house visits every other month to all households in one intervention and one control neighbourhood. Potential participants were pregnant women at least 18 years old living in the neighbourhood from May 2009 to September 2010. Recruiters obtained consent-to-contact and then scheduled transport to a research site for interviewers to obtain informed consent and a baseline assessment. Transportation was also provided for the post birth interviews at 2 weeks post-birth, 6 months and 18 months. Pregnant women were recruited at an average 26 weeks of pregnancy (range, 3–40 weeks). Only 2% of pregnant women refused participation.

**Figure 1:**
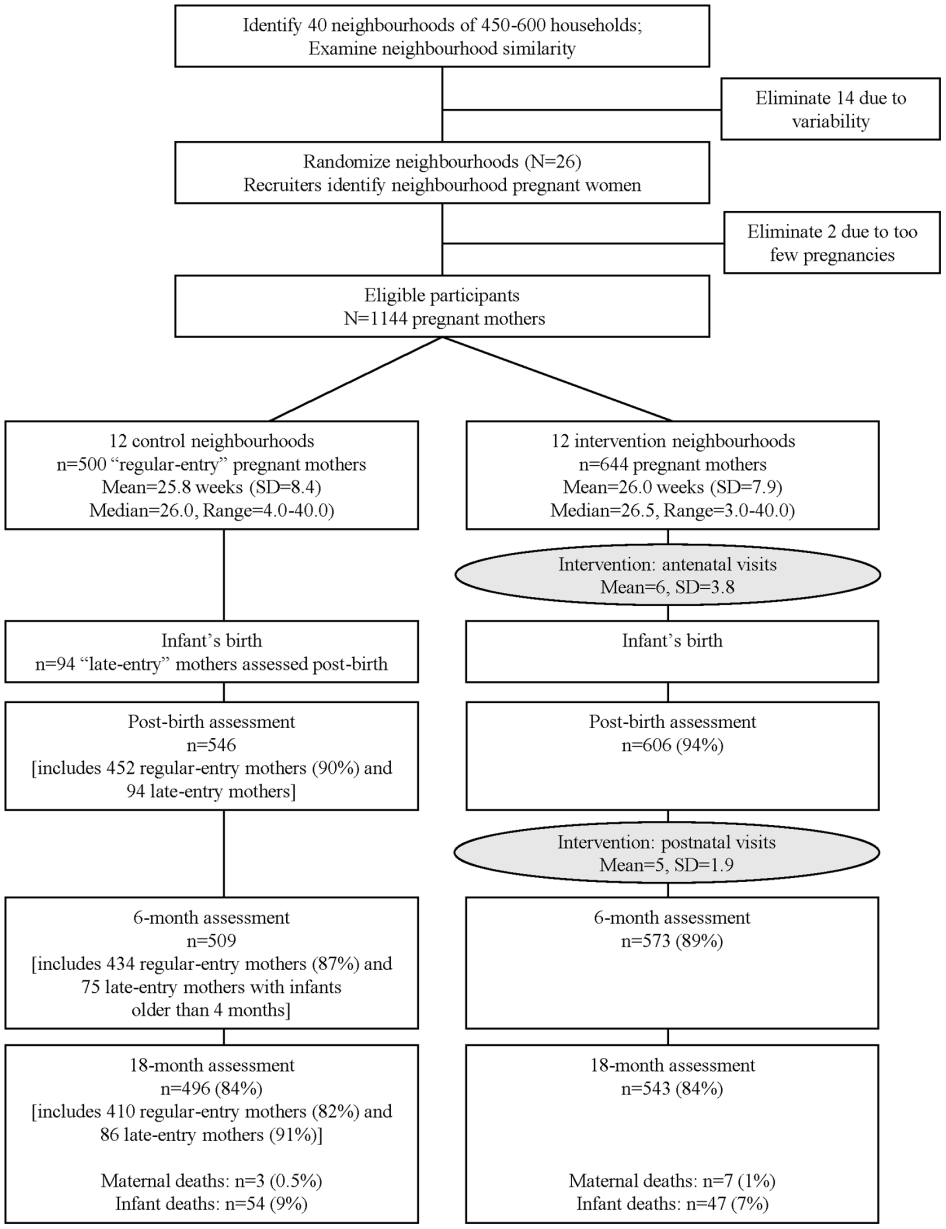
Movement of participants through the trial at each assessment point for mothers in the control and the intervention arms.

Figure 1 summarises participant flow through the study. We assessed 1238 women at baseline. Assessments were conducted post-birth at two weeks (92%; mean = 1.9 weeks; SD = 2.1 weeks; median = 1.1; range = 0.1–14.9); six months (88%; mean = 6.2 months, SD = 0.7; median = 6.0; range = 4.2–11.7); and 18 months (84%; mean = 19.1 months; SD = 3.0; median = 18.0; range = 13.6–34.4). All assessments were completed by 83% of mothers; 7% completed no follow-up reassessments; and 10% completed one or two reassessments. Although 84% of mothers completed the 18-month assessment, fewer infants were reassessed at 18 months, as mothers did not consistently bring their children to assessment interviews.

As described in an earlier publication [17], the neighbourhoods and pregnant women were highly similar across conditions. After initial recruitment, we appeared to have fewer pregnant women in the control clusters. Recruiters re-canvassed all households in each control neighbourhood and identified 94 additional women pregnant during the recruitment period (included in Figure 1 and follow-up rates above). These “late-entry” controls (16% of the control sample) were from 10 of the 12 control neighbourhoods (median of 7 late-entry participants per neighbourhood; range, 3–24). The late-entry mothers received at least two assessments. The first assessment included the questions from the baseline, post-birth, and six month interview, and abstracted data from the infant Road to Health card. The first assessment was conducted when infants were a mean age of nine months old (median = 8.9; range, 1–18 months). In addition, all late-entry mothers/infants received the 18-month assessment.

#### Assessments

We recruited, trained, and certified township women to interview participants, entering responses on mobile phones (Nokia E61i and 2630) programmed by Mobenzi (http://www.mobenzi.com/researcher/). Interviewers recorded infants' physical and developmental status, and gathered data from the infant's government-issued Road-to-Health card. Supervisors monitored and gave feedback on the data quality weekly. Data collection concluded in October 2012.

### Measures

HIV-related prevention included maternal HIV status (both self-reported and indicated on the infant Road to Health card) and disclosure of serostatus to partners; asking partners to test for HIV; and consistent condom use (on 10 of the last 10 sexual encounters). Among women living with HIV (WLH), managing one's health and stopping transmission to others requires knowing one's CD4 cell count (or not), adherence to antiretroviral medications (ARV) over the last week, and complete regimens to Prevent Mother-to-Child Transmission (PMTCT). To PMTCT, mothers must adhere to ARV starting from week 28 of pregnancy; take ARV during labour; provide ARV to infants post-birth; test infants for HIV and retrieve results at 6 weeks post-birth; use one feeding method (either breastfeeding or formula) for the first 6 months; and exclusively breastfeed.

Child health status was assessed by LBW (<2500 grams) and by converting infant anthropometric data (collected from birth records and growth monitoring) to z-scores based on the World Health Organisation's (WHO) age-adjusted norms. A z-score below -2 standard deviations was considered a serious health deficit [18]. Mothers reported the length and exclusivity of breastfeeding and risky alcohol use [19]. The features of a Fetal Alcohol Spectrum Disorder (FASD) were screened and assessed in two stages at 18 months [20]. The Bayley Scales of Infant and Toddler Development were administered at 18 months, with age-adjusted scores on cognitive and motor development [21].

Healthcare was assessed by self-reports of antenatal clinic visits, post-birth complications (heavy vaginal bleeding, malodorous discharge, fever, persistent cough, breast infection), and tuberculosis (TB) testing. Immunisation data were extracted from Road-to-Health cards.

Depressive symptoms were reported on the Edinburgh Postnatal Depression Scale (EPDS), using a cut-off of >13 to indicate depressed mood [22], [23].

Social networks' size and frequency of contact and paternal acceptance of the child were self-reported. Receipt of the government child grant was documented.

### Control and intervention conditions

#### Control arm

Standard clinic care in Cape Town is accessible and provides free HIV testing, dual regimen therapies for WLH, consistent access to milk tins (formula), TB and CD4 cell testing, co-trimoxazole for infants until HIV testing, HIV polymerase chain reaction (PCR) testing for infants at six weeks, postnatal visits at one week, treatment for WLH, and HIV testing for partners of WLH (http://www.westerncape.gov.za/eng/directories/services/11500/6389). In our sample of women who gave birth in Cape Town, approximately 79% of women gave birth in a hospital, 20% gave birth at a non-hospital facility, and 1% gave birth at home.

#### Intervention arm

In addition to clinic care, CHWs provided home visits to participants. CHWs were women with 10^th^–12^th^ grade education around 40 years old (range 34–59) who were trained for one month in cognitive-behavioural change strategies and roleplaying. They also watched videotapes of common situations that CHWs might face. CHWs were women selected to have good social and problem solving skills, having raised healthy children through their own coping skills, and were trained to provide and apply health information about general maternal and child health, HIV, alcohol use, and nutrition to township women. CHWs were certified and supervised biweekly with random observations of home visits.

### Procedures

CBW from Philani implemented the intervention. Eight health messages were delivered regarding a healthy pregnancy, HIV/TB testing and PMTCT, reducing alcohol use and malnutrition, and encouraging breastfeeding, with the aim to deliver these messages in at least four antenatal visits and four post-natal visits within the first two months of life [16]. The intervention dose delivered (i.e., the number of home visits, the visit duration, and content) by CHWs was monitored by CHWs' entries on mobile phones that included a time stamp and summary visit reports. On average, CHWs made six antenatal visits (SD = 3.8), five postnatal visits between birth and two months post-birth (SD = 1.9), and afterwards about 1.4 visits/month (range: 0.1–6.4 visits/month). Sessions lasted on average 31 minutes each.

### Analyses

We first looked for significant differences in baseline demographics between conditions at baseline and among those re-assessed (or not) at post-birth, six and 18 months. To control for multiple comparisons and measure the intervention's overall effect on well-being, our primary analysis of the intervention's impact was conducted using one overall test which compared 32 different outcomes simultaneously. On many of the outcomes, almost all mothers would have accomplished the task, without an intervention (e.g., immunize their children). The potential benefits of an intervention are relatively small for such outcomes. Comparing 32 outcomes, chance would lead one to observe up to three significantly different outcomes between the control and the intervention conditions. The binomial test evaluates the number of significant differences between the control and intervention conditions to determine if there is a significant overall difference between conditions. Thus, a binomial test evaluated the number of significant effects favouring the intervention among 32 correlated binary outcomes tested at a one-sided, upper-tail alpha = 0.025 (performed in R, version 2.11.1; please see Appendix S1 for analysis details).

Exploratory analyses compared individual measures between intervention and control at a two-sided alpha = 0.05 using logistic random effects regression models adjusting for neighbourhood clustering in SAS PROC GENMOD (version 9.2; SAS Institute Inc., Cary, North Carolina, USA). As the binomial test was the primary analysis, we considered our analyses of individual outcomes to be exploratory and retained the model p-values in lieu of further multiple-testing adjustments.

To examine if early intervention impact was sustained for the 12 outcomes that were created by combining data from multiple time points (ex: “Discussed HIV status with sexual partner at six and 18 months”), a second exploratory analysis compared these measures between intervention and control using 18-month data only, using the regressions described above.

An exploratory analysis examined the as-treated outcomes, investigating the association between each outcome and the number of CHW visits received (using the regressions described above). We included the number of visits between each assessment point *as a covariate* in order to systematically control for the number of home visits. Thus, post-birth outcomes were a function of the number of antenatal visits; six month outcomes were a function of postnatal visits between birth and the six month assessment; and 18-month outcomes were a function of the number of home visits between six and 18 months post-birth. By definition, women in the control arm had zero visits. Intervention mothers who received zero CHW visits were excluded from the as treated analysis (3%, n = 18).

Late-entry participants' data were included in all analyses. Based on their age at the late-entry assessment, data from infants of late-entry mothers were split between post-birth (0–4 months old, n = 19) and 6-month (>4 months old, n = 75) outcomes. Overall, results were similar whether or not late-entry participants' data were included; results are available upon request.

## Results

### Sample description

As previously reported [15], [16] and summarised in Table 1, mothers in the two arms did not differ significantly at baseline on outcome-related measures, neighbourhood matching criteria, demographic characteristics, risk history, or general health. Among the subset of non-primiparous women, control mothers reported more previous births than did intervention mothers. The baseline measures of mothers who were successfully reassessed at each assessment, compared to those not interviewed at each assessment, were not significantly different with one exception: mothers reassessed at 18 months were about a year older at the baseline assessment than mothers not reassessed. There were no serious study-related adverse events.

**Table 1:** Baseline characteristics of sample (N = 1238) summarized by intervention arm: intervention (N = 644) vs. control (N = 594).^1^

	Intervention (N = 644) n (%)		Control (N = 594) n (%)		Total (N = 1238) n (%)		P-Value 2	
Demographic Characteristics								
Mean age (SD)	26.5	(5.5)	26.3	(5.6)	26.4	(5.5)	0.78	
Mean highest education level (SD)	10.3	(1.8)	10.3	(1.8)	10.3	(1.8)	0.64	
Married or lives with partner	377	(58.5)	324	(54.6)	701	(56.6)	0.52	
Ever employed	129	(20.0)	104	(17.5)	233	(18.8)	0.34	
Monthly household income >2000 Rand	280	(45.6)	279	(48.1)	559	(46.8)	0.48	
Formal housing	197	(30.6)	191	(32.2)	388	(31.3)	0.96	
Water on site	333	(51.7)	327	(55.1)	660	(53.3)	0.98	
Flush toilet	340	(52.8)	343	(57.7)	683	(55.2)	0.92	
Electricity	569	(88.4)	543	(91.4)	1112	(89.8)	0.84	
Mother hungry past week	312	(48.4)	301	(50.7)	613	(49.5)	0.35	
Children hungry past week	175	(27.2)	185	(31.1)	360	(29.1)	0.05	
Maternal Health								
Mean weeks pregnant at assessment (SD)	26.0	(7.9)	25.8	(8.4)	25.9	(8.1)	0.71	
Non-primipara	422	(65.5)	394	(66.3)	816	(65.9)	0.71	
Mean number of live births (SD)	1.5	(0.9)	1.7	(1.1)	1.6	(1.0)	0.01	*
Antenatal clinic appointment	504	(78.3)	376	(75.2)	880	(76.9)	0.33	
Tested for TB, lifetime	206	(32.0)	210	(35.4)	416	(33.6)	0.23	
Test positive TB, lifetime	53	(8.2)	50	(9.4)	103	(8.8)	0.44	
Mental Health								
EPDS>13	238	(37.0)	195	(32.8)	433	(35.0)	0.27	
HIV and Reproductive Health Behaviour								
Sexual partner, past 3 months	580	(90.1)	522	(87.9)	1102	(89.0)	0.28	
Knowledge of partner HIV status			0.52					
Partner HIV+	46	(7.9)	50	(9.6)	96	(8.7)		
Partner HIV-	325	(56.0)	296	(56.7)	621	(56.4)		
Partner serostatus unknown, or no response	209	(36.0)	176	(33.7)	385	(34.9)		
Request partner HIV test	391	(82.5)	355	(83.1)	746	(82.8)	0.79	
Ever tested for HIV	590	(91.6)	550	(92.6)	1140	(92.1)	0.57	
Received HIV test results	584	(99.0)	547	(99.5)	1131	(99.2)	0.40	
Women living with HIV	149	(25.5)	146	(26.7)	295	(26.1)	0.65	
Mean number of people disclosed to (SD)	3.8	(4.5)	5.0	(7.2)	4.4	(6.0)	0.10	
Sexual partner, past 3 months	127	(85.2)	125	(85.6)	252	(85.4)	0.95	
Disclosed to partner	99	(73.9)	105	(82.7)	204	(78.2)	0.14	
Knowledge of partner HIV status			0.26					
Partner HIV+	42	(33.1)	50	(40.0)	92	(36.5)		
Partner HIV-	13	(10.2)	17	(13.6)	30	(11.9)		
Partner serostatus unknown, or no response	72	(56.7)	58	(46.4)	130	(51.6)		
Alcohol								
Drank any alcohol, month prior to pregnancy discovery	155	(24.1)	129	(25.8)	284	(24.8)	0.59	
AUDIT-C>2, month prior to pregnancy discovery	113	(17.6)	101	(20.2)	214	(18.7)	0.32	
Drank any alcohol after pregnancy discovery	56	(8.7)	49	(9.8)	105	(9.2)	0.54	
AUDIT-C>2, after pregnancy discovery	41	(6.4)	24	(4.8)	65	(5.7)	0.39	
Drank any alcohol, anytime during pregnancy	172	(26.7)	154	(25.9)	326	(26.3)	0.81	
Low Birth Weight (LBW)								
Previous LBW infants, among non-primiparous mothers	61	(14.5)	69	(17.5)	130	(15.9)	0.12	

1 Sample size for controls includes 500 regular-entry controls and 94 late-entry controls.

2 P-values from linear (continuous variables), logistic (binary), or multinomial (categorical, >2 levels) random effects regressions, adjusted for neighbourhood clustering.

*p<0.05.

### Outcome measures

Table 2 presents the comparison of control and intervention on the panel of 32 outcome measures. The intervention had significantly better outcomes over 18 months on 7 of 32 measures, resulting in significantly better overall maternal and infant well-being for the intervention compared to the control using the binomial test (average correlation = 0.2, p = 0.008).

**Table 2:** Post-birth, 6-month, and 18-month health and well-being outcomes among all participants (N = 1157), summarized by intervention arm: intervention (N = 608) vs. control (N = 549).^1^

	Intervention (N = 608) n (%)		Control (N = 549) n (%)		Estimated odds ratio, intervention vs. control, (95% CI)^2^		2-sided p-value^2^	1-sided, upper tail p-value^2^	
HIV-related prevention strategies									
*Among mothers with a current sexual partner* 3									
Discussed HIV status with sexual partner at 6 and 18 months	343	(67.3)	302	(64.9)	1.11	(0.85, 1.44)	0.442	0.221	
Asked sexual partner to test for HIV, post-birth and at 6 and 18 months	363	(59.8)	316	(58.8)	1.04	(0.87, 1.25)	0.645	0.323	
Used a condom 10 of the last 10 times had intercourse at 6 and 18 months	180	(35.3)	137	(29.5)	1.25	(1.05, 1.50)	0.014	0.007	*
*Among HIV+ mothers* 4									
Mother knows last CD4 cell count at 6 months	145	(89.5)	130	(92.9)	0.51	(0.30, 0.89)	0.017	0.992	
18-month medication doses past 7 days: none missed, all correct time and circumstances	55	(83.3)	52	(80.0)	1.40	(0.58, 3.37)	0.451	0.225	
*PMTCT*									
Mother took azidothymidine (AZT) prior to labour, or full-ARVs^PB^	169	(94.4)	149	(93.7)	1.08	(0.42, 2.80)	0.868	0.434	
Mother took AZT during labour, or full-ARVs^PB^	164	(91.6)	147	(92.5)	0.87	(0.39, 1.95)	0.741	0.630	
Mother took nevirapine (NVP) tablet at onset of labour, or full-ARVs^PB^	166	(92.7)	142	(89.3)	1.52	(0.70, 3.31)	0.291	0.146	
Infant given NVP syrup within 24 hours of birth^PB^	171	(95.5)	141	(88.7)	2.94	(1.41, 6.12)	0.004	0.002	*
AZT dispensed for infant and medicated as prescribed^PB^	172	(96.1)	142	(89.3)	2.95	(1.12, 7.73)	0.028	0.014	*
Took infant to 6-week HIV PCR test and fetched results	155	(96.9)	132	(94.3)	1.80	(0.62, 5.28)	0.282	0.141	
One feeding method first 6 months: formula or breastfeeding	96	(55.8)	64	(41.8)	1.84	(1.28, 2.65)	0.001	<0.001	*
Child health status									
Birth weight ≥2500 grams^PB^	520	(90.1)	426	(87.1)	1.35	(1.00, 1.83)	0.051	0.025	
Weight-for-age z-score ≥−2 always: birth, post-birth, 6 & 18 months	540	(88.8)	465	(84.9)	1.42	(1.01, 1.99)	0.045	0.023	*
Height-for-age z-score ≥−2 always: birth, post-birth, 6 & 18 months	470	(77.3)	413	(75.6)	1.10	(0.83, 1.45)	0.519	0.259	
Weight-for-height z-score ≥−2 always: birth, post-birth, 6 & 18 months	485	(80.2)	448	(82.5)	0.86	(0.64, 1.14)	0.284	0.858	
Head-circumference-for-age z-score ≥−2 always: birth, post-birth, 6 & 18 months	527	(86.7)	460	(83.9)	1.24	(0.94, 1.64)	0.131	0.066	
Number of months breastfed exclusively>median of 3	197	(49.5)	85	(23.9)	3.08	(2.17, 4.37)	<0.001	<0.001	*
Exclusive breastfeeding first 6 months	59	(10.3)	15	(3.1)	3.59	(1.91, 6.75)	<0.001	<0.001	*
Drank no alcohol the month prior to giving birth^PB^	566	(93.6)	443	(90.8)	1.50	(0.87, 2.58)	0.144	0.072	
No risky drinking at post-birth, 6, and 18 months (AUDIT-C score ≤2)	531	(87.3)	479	(87.2)	1.00	(0.73, 1.36)	0.999	0.500	
No FASD at 18 months	422	(99.3)	385	(99.0)	1.38	(0.37, 5.15)	0.627	0.313	
Bayley cognitive composite score ≥85 (18 months)^5^	254	(92.4)	218	(89.3)	1.44	(0.80, 2.58)	0.224	0.112	
Bayley motor composite score ≥85 (18 months)^5^	267	(97.1)	235	(96.3)	1.20	(0.54, 2.64)	0.656	0.328	
Healthcare adherence									
4 or more antenatal clinic visits (4 is standard practice)^PB^	474	(82.7)	439	(82.7)	1.00	(0.74, 1.34)	0.992	0.504	
Mother free of post-birth complications through 6 months^6^	127	(20.9)	102	(18.6)	1.16	(0.86, 1.57)	0.342	0.171	
Mother tested for TB at 6 and 18 months	35	(6.1)	47	(9.0)	0.66	(0.41, 1.06)	0.087	0.957	
Number of 18-month immunizations>median of 17 (25 total)	108	(40.3)	99	(39.8)	1.02	(0.72, 1.44)	0.910	0.455	
Depression									
Not depressed at post-birth, 6, and 18 months (EPDS≤13)	357	(58.7)	350	(63.8)	0.81	(0.63, 1.05)	0.108	0.946	
Social support									
6-month number of close friends or relatives×frequency of contact>median of 16^7^	271	(47.3)	264	(51.9)	0.85	(0.62, 1.17)	0.318	0.841	
Father acknowledged infant to family between birth and 18 months	582	(96.2)	529	(96.9)	0.80	(0.38, 1.67)	0.553	0.723	
Receiving child support grant at 6 and 18 months	310	(55.7)	306	(60.1)	0.83	(0.64, 1.08)	0.167	0.916	

1 Sample size reflects participants available post-birth, at 6 months, or at 18 months (N = 1157). Exclusively post-birth outcomes are indicated using^PB^; other outcomes' assessment times are indicated in the outcome description. Sample sizes for each assessment: Post-birth assessment: intervention (N = 606), control (N = 546, including 452 regular-entry controls and all 94 late-entry controls), total (N = 1152). 6-month assessment: intervention (N = 573), control (N = 509, including 434 regular-entry controls and 75 late-entry controls with infants older than 4 months), total (N = 1082). 18-month assessment: intervention (N = 543), control (N = 496, including 410 regular-entry controls and 86 late-entry controls, both assessed using the 18-month assessment), total (N = 1039).

2 Random effects logistic regression, adjusted for neighbourhood clustering. Models for outcomes among HIV+ mothers control for baseline employment. 1-sided p-value used in the binomial test; 2-sided p-value used in the secondary analysis of individual outcomes.

3 Measures assessed for mothers with a current sexual partner: Post-birth through 18 months: intervention (N = 607), control (N = 537), total (N = 1144); 6 through 18 months: intervention (N = 512), control (N = 469), total (N = 981).

4 Measures assessed for HIV+ mothers: intervention (N = 185), control (N = 170), total (N = 355).

5 Bayley assessment: intervention (N = 275), control (N = 244), total (N = 519).

6 Post-birth complications include heavy vaginal bleeding, malodorous discharge, fever, persistent cough, and breast infection.

7 Median number of close friends or relatives: 2. Median frequency of contact in past month: 7.

*1-sided, upper tail p<0.025.

The analysis of individual outcomes identified significant treatment differences in HIV-related prevention strategies and child health status. More intervention mothers used condoms consistently than control mothers (OR = 1.25; 2-sided p = 0.014). Infants of intervention WLH were more likely to receive nevirapine at birth (OR = 2.94; p = 0.004) and azidothymidine post-birth (OR = 2.95; p = 0.028). Intervention WLH were significantly more likely to report using one feeding method for the first 6 months post-birth than control WLH (OR = 1.84; p = 0.001). Over 18 months, intervention mothers were more likely to have infants with weight-for-age z-score ≥−2 (OR = 1.42; p = 0.045), breastfeed their infants more than the median number of months (OR = 3.08; p<0.001), and exclusively breastfeed their infants for 6 months (OR = 3.59; p<0.001). Intervention mothers tended to have fewer LBW infants (birth weight ≥2500 grams) than control mothers (OR = 1.35; p = 0.051). There were no significant differences on measures of healthcare, risky alcohol use, depression or social support.

For the 12 outcomes that were created by combining data from multiple time points, we did not find any significant differences between the intervention and the control arms when we analysed these measures using the 18-month data only (available upon request).

Each of the aforementioned outcomes was also significantly related to the number of home visits received. That is, the more home visits made by CHWs, the larger the effect sizes were on maternal and infant outcomes. In addition, the as-treated analyses found three significant differences that were related to the number of visits conducted, even though intention-to-treat analyses did not indicate a significant difference. CHW visits were significantly associated with higher odds of infant birth weight ≥2500 grams (OR = 1.07; p = 0.012), infant 6-month head-circumference-for-age z-score ≥−2 (OR = 1.09, p = 0.017), and infant cognitive composite score on the 18-month Bayley assessment ≥85 (OR = 1.02, p = 0.048).

## Discussion

The Millennium Development Goals are unlikely to be met by most LMIC, emphasising the need for effective interventions for mothers and infants living in poverty [1]. Maternal and child health in LMIC is commonly impaired by the cumulative effects of poverty and related deficits attributable to infectious diseases, malnutrition, and maternal behaviours such as alcohol use [1], [3], [15]. CHWs typically aim to improve a single targeted outcome, rather than delivering multi-foci interventions, and often are placed in health care settings [24]. There is evidence of significant benefits of single purpose CHWs, but barriers to scalability are substantial [25], [26]. Although repeatedly shown efficacious in the US [6], [7], [10], this is the first African study to evaluate a home visiting model that covers infectious disease, nutrition, and maternal caretaking concurrently.

Similar to other home visiting programs [8], [9], there were improvements in maternal adherence to a number of health regimens and infant outcomes in response to the home visits by CHWs. Perhaps the most important outcome is the reduction in the rate of LBW infants. Low birth weight carries lifelong consequences [27], especially in LMIC [28]. Prematurity and poor intrauterine growth are the primary reasons for having a LBW infant [29]. The CBW from Philani are trained to encourage pregnant women's health in multiple ways that would result in fewer LBW infants: they know risk indices for prematurity and encourage pregnant women to go immediately to antenatal care when the risk factors emerge; they check that mothers are taking folic acid and iron pills routinely; they encouraged antenatal care; they provide psychosocial support; and they encourage all mothers to test for HIV/TB. An earlier publication [17] documented that intervention mothers were significantly less likely to use alcohol during pregnancy; alcohol use is directly related to having LBW infants. Each of these behaviours by a CBW is likely to reduce the risks of having a LBW infant. These strategies are similar to those used in high income countries [27]. Rather than having CBW trained to deliver one specific health message, CBW trained as generalists who address a cluster of issues related to pregnancy promote an important benefit for infants, namely a normal birth weight.

Mothers also increased breastfeeding and HIV-related protective actions with adult partners. Infant outcomes in weight-for-age improved as well. The size of the effect was significant, but leaves substantial room for improvement. Similar to other RCT of breastfeeding interventions [30], consistent breastfeeding for the first six months of life was only 10.3% in the intervention group; however, this represents a three-fold increase over the control arm. A randomized controlled trial encouraging exclusive breastfeeding in six African countries reported similar results [30]. Although condom use was still too low for a clinical impact on HIV transmission, consistent condom use was 6% greater (OR = 1.25) with the intervention. Overall the intervention significantly improved maternal caretaking in multiple domains.

Each of the significant outcomes in our intention-to-treat analysis also was significant in the as-treated analysis. If the program were to be scaled up, the results likely would mirror the results in the intention-to-treat analyses. Notably, the number of home visits also was significantly related to children's cognitive development on the Bayley Scale at 18 months, having fewer LBW infants, higher birth weights overall, and larger head circumferences at six months. The finding of better cognitive development on the Bayley scales is particularly important, whereas benefits to measures of infant physical health were not sustained until 18 months. This RCT was designed to evaluate the intervention's impact over the first 18 months of life. Our primary analysis found significant benefits over the entire 18 month follow-up period. However, when the outcomes at only the 18 month assessment were examined, the benefits were not significantly different between the RCT's two arms, except in the as-treated analyses. To sustain efficacy, it may be necessary to continue home visits beyond the postpartum period.

Recruitment of pregnant women by canvassing household-to-household proved to be a limitation. Although the samples were highly similar across matched pairs of neighbourhoods, 16% (n = 94) of mothers in the control arm were recruited late in pregnancy or in the first months following childbirth. With or without these women in the analyses, the result of the RCT is the same. However, external validity is higher when they are included; therefore, we added the late-entry women. Fortunately, 93% of women completed at least one follow-up assessment and 83% of the sample completed all assessments. In addition, there was one selection effect across the four assessments: mothers not reassessed at 18-months were a year older at recruitment. It is also possible that self-reports for behaviours may have resulted in socially desirable answers for some outcomes. We believe, however, that the significant effects are unlikely to be attributable to reporting bias. Growth measures, health visits, immunisations, and HIV-related health behaviours are recorded on the infants' Road to Health card, similar to the documentation of the child grant.

We expected that there would be ceiling effects on many outcomes, and indeed more than 90% of participants in both arms reported positive outcomes for 8 of the 32 measures. This reflects partly the good clinical services available in Cape Town, which would be unusual in most of Africa and even South Africa. At baseline, 91% of pregnant Cape Town women were tested for HIV and received their results; this contrasts to 61% in Eastern/Southern Africa typically [31]. Among the PMTCT measures for which there was room for improvement (i.e., fewer than 90% of mothers completed the task), our intervention was better than the control in 3 of 4 outcomes, and had twice the odds of completing all behaviours needed to PMTCT [17].

Home visiting long has been demonstrated efficacious when mounted by professionals [7]–[9]. However, the next phase of research must identify sustainable and scalable models utilizing CHWs. We did not closely collect cost data at each phase of the implementation. Philani has been operating since the 1990s. The salaries paid by Philani and the CHWs' experience (typically elementary education, no previous jobs) are similar to the South African government's guidelines for paraprofessional CHW [32].

The mean of 11 CHW visits in this trial is substantially more intensive than most vertical, single-disease-targeted interventions. Regular visits were typically stopped by about 6 months post-birth, with check-ins only once every six months after that. This appears to be too small a dose for this intervention. The failure to find additional benefits from 6–18 months post-birth, when only analysing the 18 month outcomes, suggests that to sustain significant benefits, home visiting must be sustained for several years. We anticipate that home visiting must be routine through the first five years of life, for the parenting challenges shift dramatically as the children age and families have received little preparation for readjusting their parenting behaviours, beliefs, and expectations.

This intervention features a number of innovations. Typically, home visiting interventions focus on training CHWs in manualised interventions (e.g., National Registry of Evidence-based Prevention Programs [NREPP]) that must be replicated with fidelity to a set of activities and scripts, and may not fit a specific situation [33]. We selected CHWs who were positive peer deviants [34], models of pragmatic problem solving, and had good social skills. We then trained the CHWs in basic cognitive-behavioural approaches, not solely a manualised delivery. CHWs delivered specific health messages, but focused on applying the information to the mothers' daily routines. CHWs had flexibility in the sequence and duration of delivery. Implementation was routinely monitored and supervised both in person and remotely, with mobile phones [35], [36]. These innovations are atypical in programs attempting to scale evidence-based interventions, but offer a model for how prevention scientists may utilise the existing evidence-based interventions (NREPP) in a novel fashion, by training foundational skills and focusing on applying the foundational skills to a limited number of topics [2].

In addition, home visits offer a viable strategy to circumvent challenges typically associated with obtaining healthcare from clinics. Clinic appointments are difficult to schedule; waiting lines are long; transport is expensive; and mothers must coordinate their own and infants' care across multiple clinics [37]. An approach grounded in cognitive-behavioural skills, with locale-tailored content addressing local health risks, may be a strategy to explore, especially in rural communities [38].

As the South African government begins to implement a model that targets multiple domains, we have demonstrated a strategy to implement and to evaluate such an intervention. This model may be useful for countries that aim to promote task shifting from professionals to CHWs, with a solid strategy for selection, monitoring, and consistently providing outcome feedback.

## Supporting Information

Appendix S1 **Analysis details.**
(DOCX)

Checklist S1 **CONSORT Checklist.**
(DOCX)

Protocol S1 **Trial Protocol.**
(PDF)
