# Supplementary material for: A Cluster Randomised Controlled Effectiveness Trial Evaluating Perinatal Home Visiting among South African Mothers/Infants
Source: PLoS One. 2014 Oct 23;9(10):e105934. doi: 10.1371/journal.pone.0105934 (PMC4207699; doi:10.1371/journal.pone.0105934)
Supplement: Appendix S1 — Analysis details. (DOCX) [file pone.0105934.s001.docx]

Appendix: Analysis Details

Primary data analysis: binomial test for correlated outcomes

Comparisons between intervention and control on 32 binary outcomes were tested at a one-sided upper-tail alpha=0∙025 using logistic random effects regressions adjusting for neighbourhood clustering in SAS PROC GENMOD (version 9∙2; SAS Institute Inc., Cary, North Carolina, USA). All models included an indicator variable for intervention status (1=intervention, 0=control). Among WLH, we controlled for baseline employment because it differed significantly between intervention arms; among all participants there were no significant baseline differences between arms.

We can expect 32*0∙025=0∙8 significant tests (i.e. less than 1 of 32) on average if there are no differences between intervention and control. If outcomes are independent, the probability that there are 3 or fewer significant differences is 99∙2%, leading to a type 1 error of 0∙008. However, the outcomes are likely positively correlated, which does not affect the expected number of positive tests, but does affect the variance of the number of positive tests. To study the effects of global positive correlation among all outcomes on the number of positive tests assuming no intervention effect, we treated each of our 32 tests as a normal z-test (z-statistics were assumed to come from an equi-correlated multivariate normal distribution) and simulated 40,000 trials of the number of significant outcomes, for z-tests having mutual correlations rho for rho running from 0 to 0∙9 in steps of 0∙1. We declared significance for z >1∙96. Simulations were performed in R (version 2∙11∙1).

Across the 10 correlations, using a decision rule of rejecting the null of no intervention treatment effect given 4 or more significant tests of 32, the worst situation was that we reject the null with probability 0∙069 when rho=0.5. For more reasonable correlations of rho=0∙1 or 0∙2, the actual type 1 error is 0∙027 and 0∙045, respectively. We estimated the average absolute correlations among the outcomes; because variables included “true dichotomies” (e.g. “Asked partner to test for HIV”) and indicators created by dichotomising continuous outcomes (e.g. “Weight-for-age z-score ≥ -2”), we estimated both the Pearson and the tetrachoric correlations, planning to use whichever method produced higher average absolute correlations. If the correlations were found to be higher than 0∙2, we would increase the needed number of significant results from 4 to 6 of 32 before declaring the intervention’s significance. This would keep the type 1 error below 0∙05, no matter what the outcomes’ correlations.

Exploratory analyses

We tested the intervention’s impact on individual outcomes at a two-sided alpha=0∙05 using the regressions described above. For as-treated analyses, the number of visits was the independent variable, and replaced the above-mentioned indicator of intervention status. As the binomial test was the primary analysis, for our exploratory analyses we retained the model p-values in lieu of further multiple-testing adjustments.
