# Supplementary material for: A Cluster Randomised Controlled Effectiveness Trial Evaluating Perinatal Home Visiting among South African Mothers/Infants
Source: PLoS One. 2014 Oct 23;9(10):e105934. doi: 10.1371/journal.pone.0105934 (PMC4207699; doi:10.1371/journal.pone.0105934)
Supplement: Protocol S1 — Trial Protocol. (PDF) [file pone.0105934.s003.pdf]

Prev Sci (2011) 12:372–388
DOI 10.1007/s11121-011-0238-1

Philani Plus (+): A Mentor Mother Community Health Worker Home Visiting Program to Improve Maternal and Infants' Outcomes

Mary Jane Rotheram-Borus · Ingrid M. le Roux · Mark Tomlinson · Nokwanele Mbewu · W. Scott Comulada · Karl le Roux · Jacqueline Stewart · Mary J. O'Connor · Mary Hartley · Kate Desmond · Erin Greco · Carol M. Worthman · Faith Idemundia · Dallas Swendeman

Published online: 18 August 2011
© Society for Prevention Research 2011

Abstract Pregnant mothers in South African townships face multiple health risks for themselves and their babies. Existing clinic-based services face barriers to access, utilization, and human resource capacities. Home visiting by community health workers (CHW) can mitigate such barriers. The Philani Plus (+) Intervention Program builds upon the original Philani CHW home-visiting intervention program for maternal and child nutrition by integrating content and activities to address HIV, alcohol, and mental health. Pregnant Mothers at Risk (MAR) for HIV, alcohol, and/or nutrition problems in 24 neighborhoods in townships in Cape Town, South Africa (n=1,239) were randomly assigned by neighborhood to an intervention (Philani Plus (+), N=12 neighborhoods; n=645 MAR) or a standard-care control condition of neighborhood clinic-based services (N=12 neighborhoods; n=594 MAR). Positive peer deviant "Mentor Mother" CHWs are recruited from the township neighborhoods and trained to deliver four antenatal and four postnatal home visits that address HIV, alcohol, nutrition, depression, health care regimens for the family, caretaking and bonding, and securing government-provided child grants. The MAR and their babies are being monitored during pregnancy, 1 week post-birth, and 6 and 18 months later. Among the 1,239 MAR recruited: 26% were HIV-positive; 27% used alcohol during pregnancy; 17% previously had low-birthweight babies; 23% had at least one chronic condition (10% hypertension, 5% asthma, 2% diabetes); 93% had recent sexual partners with 10% known to be HIV+; and 17% had clinically significant prenatal depression and 42% had borderline depression. This paper presents the intervention protocol and baseline sample characteristics for the "Philani Plus (+)" CHW home-visiting intervention trial.

Keywords HIV · Maternal & child health · Alcohol · Nutrition · Home visiting · South Africa · Community health workers (CHW) · Paraprofessionals

Introduction

Pregnant women in South Africa face intersecting epidemics of HIV, alcohol abuse and malnutrition. South Africa has the

Thank you to the mothers and others who are participating in this work and make this research possible. This study was funded by the National Institute on Alcohol Abuse and Alcoholism (1R01AA017104). ClinicalTrials.gov registration # NCT00972699.

M. J. Rotheram-Borus · W. S. Comulada · M. J. O'Connor · K. Desmond · E. Greco · F. Idemundia · D. Swendeman
University of California,
Los Angeles, CA, USA

I. M. le Roux · N. Mbewu · K. le Roux · J. Stewart · M. Hartley
Philani Nutrition and Development Project,
Cape Town, South Africa

M. Tomlinson
Stellenbosch University,
Stellenbosch, South Africa

C. M. Worthman
Department of Anthropology, Emory University,
Atlanta, GA, USA

M. J. Rotheram-Borus (✉)
Center for Community Health,
10920 Wilshire Blvd., Suite 350,
Los Angeles, CA 90024-6521, USA
e-mail: MRotheram@mednet.ucla.edu

highest number of persons living with HIV globally (5.2 million) (UNAIDS 2007). Concurrently, it has the highest documented rate of Fetal Alcohol Syndrome (FAS) and 17% of mothers have at least one child with a birth weight under 2500 g, resulting in lifelong negative health outcomes (May et al. 2000, 2004, 2005). Clinic-based antenatal services are available but typically do not address alcohol and depression problems. However, 30% of pregnant women in South Africa do not access antenatal care (Rollins et al. 2007). Furthermore, the recognized lack of professionally trained healthcare workers, and limited time available for prevention counselling in clinic-based settings, has led to recommendations from the World Health Organization (WHO; 2008) to "task-shift" services that do not require nurse or physician expertise to paraprofessional community health workers (CHWs). This paper describes a CHW-delivered home-visiting intervention (i.e., the "Philani Plus (+)" Intervention Program), its rationale, and the baseline sample characteristics of the pregnant mothers-at-risk (MAR) for HIV, alcohol, mental health, and nutrition problems in South African township neighborhoods who were recruited to participate in a community-randomized trial of the intervention.

In the United States and elsewhere, home-visiting programs for new mothers have been implemented and evaluated over the past 30 years, finding positive impacts on both short- and long-term health and social-emotional development outcomes (Gomby et al. 1999; Olds et al. 2007; Sweet and Appelbaum 2004). These programs target families from a variety of different populations, although a majority are from low-income areas and primarily include at-risk mothers. Home-visiting programs typically target pregnancy outcomes, child social-emotional and cognitive development, child abuse, and positive parenting. Reviews and meta-analyses have found overall positive impacts of these programs; however, significant variations in population, intervention content, outcome measures, and training of the home visitor in particular make it difficult to identify which specific components of home-visiting programs are most responsible for efficacy (Gomby et al. 1999; Sweet and Appelbaum 2004).

The paradigmatic model is for nurses to deliver home-visiting interventions for at-risk families, which is based on evidence from a randomized trial in the U.S. that found no significant effects for a paraprofessional-delivered home-visiting intervention compared to a nurse-delivered program or a control condition (Olds et al. 2002). However, it is not feasible to implement nurse-delivered home visits broadly, particularly in South Africa and other low- and middle-income countries, as well as in the U.S., due to the global shortage of professionally trained nurses and sky-rocketing healthcare costs or limited healthcare budgets for many countries (Chaguturu and Vallabhaneni 2005). Thus, there is broad agreement and ongoing trends toward shifting clinical and prevention tasks to paraprofessionals (i.e., CHWs). Globally, there are more than 40 million CHWs (Lewin et al. 2008). CHW programs have been efficacious in reducing infant mortality (Bang et al. 2005; Manandhar et al. 2004; Sazawal and Black 2003). CHW-delivered home visits have also been found to be a very effective strategy for HIV testing (Mermin et al. 2004; Were et al. 2003), improving infant and child health (Bang et al. 2005; Sweet and Appelbaum 2004), and improving maternal and child nutrition (le Roux et al. 2010, 2011). Utilizing paraprofessional CHWs may be the only realistic and feasible strategy to scale-up and broadly disseminate prevention services such as home-visiting interventions for new mothers and children (WHO 2008). Unfortunately, the effectiveness of CHW has been unreliable in each large-scale replication trial due to inconsistency in selection, training, monitoring, supervision, and support for CHWs (Haines et al. 2007; Tomlinson 2010; Walley et al. 2008).

Background on the Philani Nutrition Intervention Program in South Africa

Philani is a non-government organization (NGO) operating in 150 township neighborhoods that focuses on improving a child's nutritional status. Philani identifies potential neighborhood CHWs on the basis of being "positive peer deviants"[1] (Berggren et al. 1984; Marsh et al. 2004; Sternin et al. 1998). Mothers in the township neighborhoods, who are positive role models in that their children are thriving in the community, are recruited and trained as paraprofessional "Mentor Mother" CHWs to make home visits to monitor and support infant and child nutritional status and development, refer to clinic care, and become sources of ongoing social support to their neighborhood peers. Mothers whose children thrive typically have good social skills, an ability to develop caring social relationships, and a sense of pragmatism. These Mentor Mothers bond with pregnant women and can problem solve common challenges facing township families with knowledge of and experience with the community's risks and available resources.

Rather than case-finding from a clinic list, Philani's Mentor Mothers draw their case load from a geographically-defined area; that is, a neighborhood. Philani's Mentor Mothers systematically visit all mothers in a neighborhood to identify those most at risk for health problems and who might not present at clinics. Visiting all mothers in a neighborhood simultaneously builds a supportive network and social capital within an area while minimizing stigma

[1] The word "deviant" is in a positive direction from the norm of the township mothers. The theory utilized is labeled the "Theory of Positive Peer Deviants."

associated with services that narrowly target the highest risk individuals in a community.

Perhaps most important, a stable funding stream exists for the Mentor Mother program: the Integrated Management of Childhood Illness (IMCI), which is also endorsed by the WHO (2010). Nationally in South Africa, community workers are hired in each community for IMCI, but receive little or no training. There are no standards other than referring women for clinical services and their child grant. A community worker is typically expected to refer at least nine women monthly to local clinics on the first day of the month, an easily accomplished goal.

The original Philani Intervention Program has a 30-year history of successful, non-stigmatizing, and sustainable home-based support for pregnant women and malnourished children. Program evaluations of the Philani program have found increased weight of malnourished children (le Roux et al. 2010), higher rates of child rehabilitation, and faster rehabilitation of children over 1 year (le Roux et al. 2010). However, township mothers and children face many other significant challenges in addition to malnutrition, such as HIV, alcohol abuse, and poor mental health that could also be addressed during CHW home visits.

Background & Rationale for the Philani Plus [+] Program

The Philani Plus (+) Intervention Program was conceived to integrate into the existing Philani Intervention Program content and activities to address HIV, alcohol, mental health, and healthy daily routines for a variety of chronic conditions and stressors. The current randomized control trial (RCT) is based on the pre-existing Philani nutrition program, with expanded content, enhanced standardization, and systematic implementation. The Mentor Mothers have been provided training, materials, and skills to address major community health challenges of HIV, TB, malnutrition, and alcohol use. We anticipate that selecting positive peer deviants from the neighborhoods to serve as CHWs will support the effectiveness of the program, in addition to training and continuous supervision and support.

Pregnancy The timing of pregnancy creates opportunities that are unique not only from the perspective of the mother's and infant's biology, but also from a behavior change perspective. Coping with and stopping risky actions regarding HIV, TB, alcohol and nutrition are habitual behaviors. Verplanken and Wood (2006) have demonstrated that it is far easier to shift behaviors during a life transition, such as a child's birth. Relationships become reordered, sleeping and eating arrangements often are shifted, and resource allocations change. Pregnancy is a window of opportunity to shift health habits and daily routines disturbed by pregnancy and the infant's arrival.

Clinical Care Although clinics and IMCI services are available in South African township neighborhoods, pregnant women face many barriers to accessing and utilizing available clinical services that the Philani and home-visiting interventions aim to address. For example, accessing care often requires coordinating among several different clinics (Rotheram-Borus et al. 2004a). In Cape Town, an HIV+ pregnant mother would need to access care in four separate health care sites within a 4-month period: 1) antenatal care; 2) well-baby care; 3) HIV testing of fathers; and 4) an HIV clinic for the HIV+ mother. If infected with TB, she would need to visit yet another clinic for TB care. In addition, there are predictable stressors and logistical challenges associated with seeking care, such as transportation, childcare, and uncertain wait times at clinic visits. Navigating these hurdles to consistently access clinic-based treatment and care requires problem-solving skills and knowledge of the healthcare system, skills and knowledge that Mentor Mothers are selected for and then trained in how to systematically teach other mothers and families.

Once logistical hurdles are overcome, translating medical providers' recommendations into a healthy daily routine is not easy and requires more problem solving and support than is typically available in one or two antenatal clinic visits (Rollins et al. 2007). Prevention programs usually target one specific behavior for change even though many health behaviors are linked; for example, nutrition and alcohol use. Instrumental and emotional support in prevention settings has been consistently linked to improved outcomes (Durantini et al. 2006) and infant health specifically (Morrison et al. 2005), and yet is not easily delivered in clinical settings. Home visiting by Mentor Mothers is more accessible to families than clinic visits and Philani Plus (+) supports ongoing contacts with mothers and families. Furthermore, Philani home visiting is acceptable to township mothers and families because it is identified as a nutrition program and does not have the stigma associated with HIV-identified programs, for example. Being visited does not signify that the family has trouble with topics consistently addressed by Mentor Mothers: HIV, alcohol, pregnancy, depression, and nutrition.

HIV About 26% of township mothers in Cape Town are Mothers Living with HIV (MLH). HIV+ mothers have challenges caring for their own compromised health, as well as that of their baby. These mothers must engage in at least 15 different behaviors to keep their babies safe from HIV; for example, taking AZT during the last weeks of pregnancy; disclosing serostatus to the nurse at childbirth; taking NVP during labor; taking AZT and giving it to her baby; using a single feeding method for 6 months, preferably only breastfeeding; getting the child HIV tested at 6 weeks; receiving the HIV test results for the child; and going to a

clinic to monitor for her own health. These behaviors need ongoing support, and are not easily addressed in a clinic visit.

Alcohol South Africa has the highest per capita alcohol consumption rate, including hazardous alcohol use during pregnancy (May et al. 2005). Alcohol use is higher among mothers in Coloured than in Black townships (30–50% vs. 15–20%; Dermen and Cooper 2000). Alcohol use disinhibits sexual behavior, and alters judgment, memory processes, and self-regulation of arousal (Morojele et al. 2006). Alcohol abuse is associated with an increased number of sexual partners (Morojele et al. 2006), which increases risks for HIV and unplanned pregnancy. In South Africa, alcohol use is also responsible for 60% of automobile accidents, more than 75% of homicides, 50% of non-natural deaths, 67% of domestic violence, 30% of hospital admissions, and costs the country about R9 billion annually— or 1.3 billion $USD— (Frontline Fellowship 2007).

Alcohol and Tobacco Use Impacts on Children Children of mothers with hazardous, dependent, or harmful alcohol use have changes in body and brain morphology, demonstrate deficits in cognitive functioning, verbal fluency, executive functioning, motor development, school achievement, and experience emotional and behavioral problems (Kodituwakku et al. 2001; May et al. 2004; O'Connor and Kasari 2000; O'Connor et al. 2002). Even low levels of alcohol consumption have been shown to be related to negative developmental sequelae for babies (Riley and McGee 2005). Among alcohol users, 75% also smoke cigarettes (Bryant 2006), a behavior linked to having underweight babies. The smoking epidemic is relatively recent in Africa, yet it is estimated that 46% of pregnant Coloured women (HIV and non-HIV infected) routinely smoke (Groenewald et al. 2007). South African pregnant women are also susceptible to the harms of second-hand smoke compounded with environmental ills such as smoke from burning solid fuels and high levels of air pollution (Bloch et al. 2008).

Depression In South African townships postpartum depression rates exceed 30% (Cooper et al. 1999) and are significantly related to alcohol, unemployment, unplanned pregnancy, perceptions of the consequences of HIV, and a lack of social support. Comorbid alcohol use and depression negatively impact infant outcomes (Kelly et al. 2002), particularly infant malnutrition and stunting, in South Africa. Mothers with depressive symptoms are less nurturing when interacting with their children (Hall et al. 1998) and are most resistant to alcohol treatment (O'Connor et al. 2002).

Child Malnutrition In South Africa, 8.9% of babies have wasting syndrome and 17% of newborns have low birth weights (Sood et al. 2001). Each of these conditions is associated with maternal depression, as well as developmental delays, premature death in childhood, children becoming diabetic, and obese adults; 45–75% of township adults are obese (Barbarin 2003; Myer et al. 2004). About 12% of South African children die before their fifth birthday (Zere and McIntyre 2003). Child malnutrition is responsible for 2% of those deaths, along with 3% of childhood disabilities and long-term reduction in quality of adjusted life years (Barbarin 2003; Myer et al. 2004; Zere and McIntyre 2003). Therefore, intervening for coordinated improvements in alcohol, HIV, and child nutrition is critical. Typically, funding streams are categorical and one health worker is not expected to simultaneously address HIV, nutrition, alcohol, and mental health. The IMCI funding creates the opportunity to assign one health worker, a Mentor Mother CHW, for a single geographic area to address all of these problems.

Methods

Philani Plus (+) Intervention

Selecting Mentor Mother CHWs Mentor Mothers are identified in a threestage process: 1) referrals by local leaders and stakeholders; 2) observations of supervisory community health workers during trainings and of trainees' homes; and 3) performance during training (coming on time, keeping notes, demonstrating problem-solving ability and interpersonal competence).

Training & Supervision Training for the paraprofessional Mentor Mothers focuses on adapting existing evidence-based HIV- and alcohol-related interventions (O'Connor and Whaley 2007; Rotheram-Borus and Duan 2003; Rotheram-Borus et al. 2004a, b; Teasdale and Besser 2008) to a new South African cultural context (South Africa National Department of Health 2008). Mentor Mothers were trained over a 2-month period initially and a comprehensive intervention manual was designed. From among 40 women accepted for training, 13 were hired as Mentor Mothers. The Mentor Mothers work half-time (4 h daily), making home visits on 4 days and attending supervision 1 day weekly. Twice monthly, a supervisor attends the home visits with the Mentor Mother.

A mobile phone intervention delivery support, monitoring, and supervision system is also utilized by the Mentor Mothers who carry study-issued mobile phones with a study-specific application loaded onto it. When a mother prepares to enter a home for a visit she enters the client's identifying information into the phone and receives a confirmatory prompt as to which pre- or postnatal session needs to be delivered. Upon exiting the home, the Mentor Mother is prompted to select which of eight core intervention topics

were discussed at the visit. The visit duration is automatically recorded based on the entry and exit survey time stamps. This information is reviewed by supervisors and discussed in case consultations during the weekly supervision meetings where they discuss why certain topics were not addressed (e.g., not applicable, or no time due to other priorities) and provide decision-making support.

Intervention Protocol Four antenatal and four postnatal sessions were specifically targeted for MAR. Mentor Mothers visit MAR once every 2 weeks over 2 months to deliver the four antenatal sessions, once every 2 weeks after birth over 2 months to deliver the four postnatal sessions, and then check in with the MAR and family about once a month to deliver support as needed, including daily visits if a family is in a crisis. The home visits typically take about 20 min but can last up to 60 min if a family has multiple stressors or is in crisis.

One Mentor Mother systematically visits every home in her assigned neighborhood. She carries a scale to weigh all children under 5 years, and uses growth charts to identify malnourished children (i.e., those two standard deviations below their age-to-weight norm). All pregnant women are encouraged to attend at least four antenatal sessions at the local clinics, to take prenatal vitamins and folic acid provided by clinics, to exclusively breastfeed their children, to delay solid foods for their babies for 6 months, and to abstain from alcohol and smoking during pregnancy. Adherence to health regimens are addressed for avoiding smoking and drinking, eating healthy foods, exercising regularly, and taking vitamins and all medicine as prescribed in antenatal care. Mentor Mothers address the following specific topics: living with HIV, alcohol use, nutrition, child assistance grant and other resources, and self-care and social support, as detailed below.

1) *Mothers Living with HIV.* Strategies about universal precautions for blood spills are reviewed, encouragement to get her partner tested for HIV, consistent condom use, and strategies for coping with partner's alcohol use and multiple partnerships are addressed.

   Disclosure is a huge issue for all persons living with HIV, including pregnant women. Deciding whom, when, what, and how to disclose their serostatus often requires time, reflection, and rehearsal. The Mentor Mother provides active support to the MLH to take these steps. Within couples, antenatal testing (96% of women) leads the woman to be the first household member to be identified as HIV+. To counter any stigma (first detected is associated with beliefs about being the first infected) and to avoid the mandate for the MLH to disclose her serostatus, study women are provided referral letters asking all couples with a pregnant woman to get tested for sexually transmitted diseases with their partner concurrently (Rotheram-Borus et al. 2005). In addition, MLH usually must tell the sister/nurse at the hospital that she is HIV+. So that the MLH does not have to verbally disclose her serostatus at the hospital, Mentor Mothers give her a card to hand the nurse regarding her status: "I am HIV+, please make sure that I get NVP." The Mentor Mother also shows the MLH the Nevirapine bottle and appropriate administration method, so that she is prepared for the NVP at childbirth. The Mentor Mother also supports adherence to AZT before and after childbirth.

   MLH also need to choose a single feeding method, ideally breastfeeding. This recommendation is difficult to follow, because clinics hand out canned milk to MLH and advocate use of bottled milk. Fear of partners', mothers', and mother-in-laws' critical observations of the feeding method are significant barriers to a single feeding method. In particular, traditional healers often advocate giving babies gripe water that contains alcohol for children, and solid food is often introduced by 3 months. Consistent with South African government guidelines, we recommend that formula feeding be avoided unless the MAR has clean water on the premises, a toilet, and enough money to buy the milk on an ongoing basis, especially if clinic supplies are depleted.
2) *Mothers with Hazardous Alcohol Use*. A realistic black baby doll with features of FAS Disorder is presented on a home visit. The characteristics and life-long consequences of alcohol on babies are discussed. Mentor Mothers are taught how to assess the typical amount of alcohol being used by the pregnant woman and to equate the level of alcohol use to the development of negative behavioral sequelae in their infants. The Mentor Mother screens for alcohol use with the Derived Alcohol Use Disorder Identification Test from the National Epidemiologic Survey on Alcohol and Related Conditions and has a brief intervention script on reducing alcohol use to deliver, as needed (Dawson et al. 2005).
3) *Nutrition.* Post birth, the Mentor Mother monitors the infant's nutrition over time in all families (e.g., they carry a scale and developmental charts). The chart shows the expected height/weight for each baby, key aspects of the home environment (e.g., mother is dressed, has fed children, home is neat, children's location is monitored). This chart is updated on each home visit by the Mentor Mother and kept over 18 months by Mentor Mothers on each family. Mentor Mothers do not distribute food, except in the most dire cases, but help the mother problem solve how and where to get food.

4) *Child Financial Assistance Grant*. Since 2002, all mothers are entitled to R240 monthly, a government grant for all financially needy mothers that provides a family safety net for children. Getting the child grant requires presenting an ID card and the baby's birth certificate. Documentation of birth and ID number are often difficult for mothers and many do not access the grant; the anticipated base rate of access is 36%. Models and strategies for problem solving challenges to getting the ID card are reviewed with mothers, along with health records. Mentor Mothers are trained on "how to work the system" for securing the grant. Philani currently provides social worker back-up to mothers on a routine basis. Mentor Mothers refer the mothers to the Philani social work program, if necessary, to help complete the paper work for the child grant.
5) *Self-care & social support to prevent depression.* Mothers are encouraged to enjoy life and take time for themselves. Other messages include doing things to care for and enjoy their baby, keeping their friends close, and being receptive to help from others. Mentor Mothers are selected on the basis of knowing how to create pleasant and supportive households. Mentor Mothers share their optimism and caring with mothers on an ongoing basis and support mothers in noticing daily joys in their children and family.

Study Design

Objectives

A RCT is being conducted to test the efficacy of paraprofessional Mentor Mothers to help MAR to better meet their antenatal and postnatal health and mental health challenges. We hypothesise that MAR and infants in the intervention neighborhoods, compared to the standard care MAR, will have improved:

- Child health status (e.g., birth weights, developmental milestones);
- Healthcare utilization and health monitoring (e.g., pre- and postnatal appointments)
- HIV-related preventive behaviors (e.g., condom use, disclosure, HIV testing);
- Mental health (specifically depression)
- Social support

Study Design Overview

Neighborhoods in three Cape Town townships (n=40 neighborhoods) were identified based on housing density, type of housing (formal or informal), sources of water (on the premises/not), length of residence (based on 30 surveys of residents per neighborhood), and the number of bars (*shebeens*). We identified 24 of the 40 neighborhoods meeting all study criteria; UCLA then randomized neighborhoods into the intervention or the standard care condition. An independent assessment team from Stellenbosch University conducted in-person interviews during pregnancy, 1 week post-birth, and 6 months and 18 months post-birth evaluating MAR and their babies' health and mental health.

As shown on Fig. 1, we randomized 24 neighborhoods to the intervention (n=12 neighborhoods) or standard-of-care control (n=12 neighborhoods) conditions, recruited and assessed 1,239 pregnant MAR (n=594 control, n=645 intervention, N=51 per neighborhood), and repeated assessments at postpartum and 6 and 18 months. The enrollment criteria were: 18 years or older, less than 34 weeks pregnant at intake, living in the selected neighborhood and able to give informed consent, as judged by the interviewer (i.e., appeared able to understand questions, not actively hallucinating or inappropriate in the interview).

Collaboration

The study is a collaborative effort between the Global Center for Children and Families, University of California, Los Angeles (UCLA; M.J. Rotheram-Borus, PI), Stellenbosch University (Tomlinson, PI) and the Philani Program (le Roux, PI).

Research Ethics and Approval

The Institutional Review Board of the University of California, Los Angeles (UCLA, G07-02-022), and the Research Ethics Committee of the Stellenbosch University of South Africa has approved and monitors the study protocol over time. A four-member Data Safety and Monitoring Board (DSMB), consisting of local and international experts, monitors the implementation of the trial. National, provincial, district, and municipal health authorities approved the study and its protocol, and set the conditions of the standard care practice. The Community Advisory Board (CAB), consisting of local stakeholders, held quarterly meetings. The CAB served as liaisons between research staff and the community, advised on study policies, and were kept informed of progress.

Study Setting

Cape Town contains five major peri-urban settlements with formal and informal rudimentary housing. Unemployment in Cape Town townships is estimated at between 25% and 50% (City of Cape Town official website:

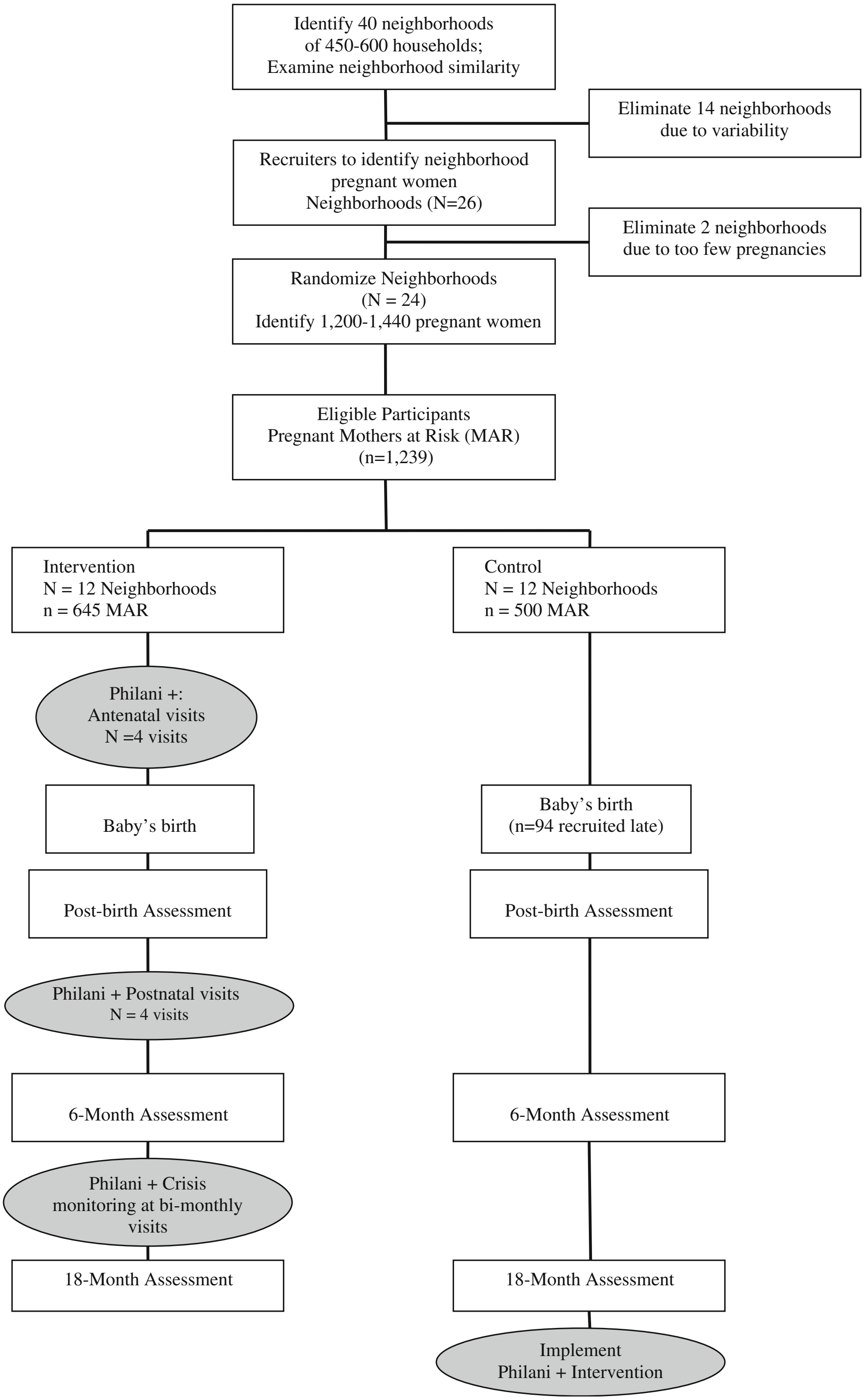

Fig. 1 Movement of participants through the trial at each major point

Economic Statistics). In total, 60% to 80% of the population have either no income or earn less than R800 per month ($111 USD). The population is young, women have a high fertility rate (3–4 children each), and most women live within 5 km of a prenatal clinic. In each area, there is sub-economic formal housing and vast areas of informal houses (shacks). Informal settlements are typically provided with tarred roads, mast lighting, water, and

water-borne sewerage services and some access to electricity and telephones. However, some informal settlements are in transit camps for people awaiting allocation of serviced sites; services are then limited to shared water outlets and bucket toilets. There are many government services and NGOs in Cape Town, as well as schools and health care services (City of Cape Town official website: local government services).

Neighborhood Selection, Matching & Randomization

We first obtained aerial maps from government offices and identified formal and informal settlements, and mapped the existing Philani intervention neighborhoods in order to ensure that no sites were selected close to the existing agency intervention work. We counted structures in each potential site from these maps. Multiple reviewers, both persons familiar and not familiar with the site, drove and walked around each neighborhood multiple times to identify comparable locales. Buffer sites were identified between each site to reduce any possibility for contamination. We identified 40 neighborhoods *without* Philani services and then matched the 40 neighborhoods into 10 community clusters of 4 neighborhoods each. The neighborhoods within matching clusters were similar in size (within 50 households), distance to the local health clinics (within 5 km or not), access to water on the premises or not, ethnic composition (majority Black or Coloured), and formal or informal settlements. Based on these activities, we narrowed the potential neighborhoods to 28 and assigned sites to groups of 4 based on similarity in the matching criteria.

Local Xhosa-speaking women also conducted 20 interviews in each neighborhood in order to ensure that the mothers that lived in the neighborhood are from similar areas in the Eastern Cape, ascertain the lengths of residence in the neighborhood, and survey housing and living characteristics. A data collector walked every street of each neighborhood and counted the number of informal and formal houses in each, as well as the number of structures such as bottle stores, shebeens, and clinics. Data were analysed using frequencies and cross-tabulations. Descriptive data such as housing type, water supply, and other infrastructure proved largely consistent with expectations derived from census data (City of Cape Town official website: 2001 Census) and 2007 satellite photography, and confirmed the majority of provisional matches in neighborhoods that were formed prior to fieldwork. However, one pair of neighborhoods was discarded because they were not sufficiently similar, and a further pair of neighborhoods was excluded because they had been vacated by the time the data collection began. The study, therefore, confirmed 12 matched pairs of neighborhoods, 24 total neighborhoods.

The 24 sites finally selected for participation comprised 450–600 households each in the Philani communities that are non-contiguous and far enough apart (at least a kilometre) to prevent cross-site contamination (e.g., with natural barriers such as roads, rivers, trash dumps). The word “neighborhood” is misleading, in that there is not an organizational infrastructure that separates these clusters of 450–600 families. The count of child-bearing women per household during pilot averaged at 1.4 according to preliminary surveys, which was lower than the anticipated 2 per household. Thus, the borders of each neighborhood were subsequently expanded slightly to ensure enough households per neighborhood to generate our sample of pregnant women for the RCT in the space of 1 year.

UCLA randomized all neighborhoods to the intervention or standard-care control conditions. Figure 2 shows the neighborhood clusters in the Endalini township and the dots reflect pregnant women that have been identified to date in each neighborhood. Each cluster is about 300 m by 300 m in size.

Standard Care Control Condition

The control condition in this study is the standard care provided in the townships, which include antenatal clinics, hospitals for delivery, primary care clinics for postnatal and well-baby care, dedicated postnatal clinics for HIV+ women and babies, and separate sites for HIV testing of male partners. Standard PMTCT (Prevention of Mother to Child Transmission) programs are provided by clinic nurses across all clinics, both those in the standard care and intervention conditions. In South Africa, standard clinic care of mothers living with HIV includes: dual therapy for PMTCT, referral to ARV for women with CD4 counts below 200 or WHO Stage 4 disease, the return of PCR test results for infants by 6 weeks of age, and co-trimoxazole for exposed infants starting at 6 weeks of age (WHO & UNICEF 2009). All Cape Town clinics distribute free milk powder at birth and milk is available to all HIV+ mothers (even though bottle feeding is not the desired behavior).

Participant Recruitment

In each neighborhood, we employ as a recruiter a mother living in the area who knows the residents. She goes house to house on an ongoing basis to identify pregnant women, obtain consent to contact, and refer pregnant women to the assessment team. A driver takes the potential participants to a centralized assessment center located at the intersection of three townships. Each recruiter covers one neighborhood in the intervention and one in the control condition. While we

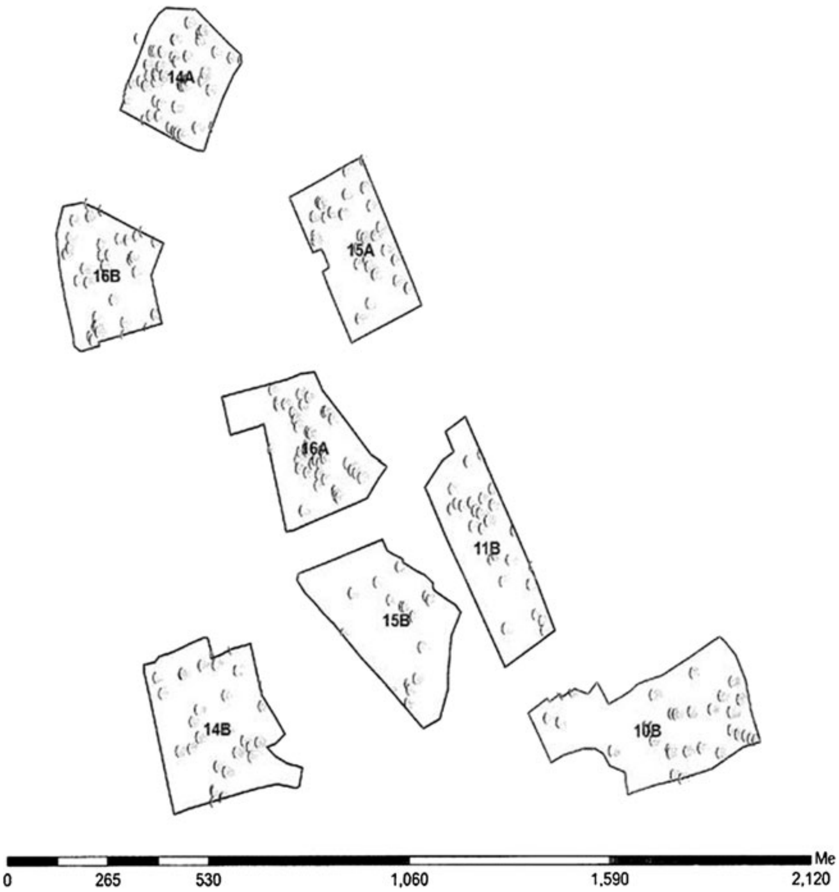

Fig. 2 Endalini township neighborhood clusters of pregnant women

do not discuss the intervention assignments of intervention or control, it is impossible for recruiters not to know in which neighborhoods the intervention is mounted. We address potential employee distortion of data by monitoring recruiters' daily progress through the neighborhood on a consistent basis. Recruiters do not collect data except for the GPS location of a potential participant's home via a study issued mobile phone.

Late Entry Participants (LEP) Recruitment in the control communities was not as thorough or timely as in the intervention communities as the Mentor Mothers provided another source of potential participant identification and referral to the assessment team. In addition, Mentor Mothers built trust in the study as they conducted their daily work in the neighborhoods, which resulted in a more thorough and rapid recruitment in the intervention neighborhoods. To achieve our goal for balanced samples and recruiting all mothers with infants in the selected neighborhoods, in the control communities we also recruited and enrolled mothers who were pregnant during our recruitment period but had already given birth before being identified and referred to the assessment team. Of the 594 mothers recruited in the control communities, 94 were late entries post-birth.

Data Collection

Mobile phones are used for collecting all interviews by field interviewers assessing the participants over time, as well as for the intervention delivery. Programming and bench testing of mobile phones for collection of assessment interviews was conducted for several months. Data are collected on mobile phones running a survey software package (Tomlinson et al. 2009). This platform allows the phone to be used to collect and upload numeric, voice and text data. Two models of phone are used in the study—the Nokia E61i (a business phone similar to a PDA) used by the assessment team, and the Nokia 2630 (an inexpensive candy-bar phone) used by the Mentor Mothers for intervention monitoring.

Initial training for the assessments covered practical aspects of phone navigation, checking for software updates, use of the software, and uploading data to the central server. Staff then spent 2 weeks familiarizing themselves with the baseline survey on the phone. During this time multiple tests were run to ensure that all data entered on the phone were uploaded and that the response entered on the mobile corresponded to the value stored in the database. Mentor Mothers use a text-messaging system for intervention monitoring (described below) that required less

training since all were familiar with text-messaging on mobile phones.

Three types of data are consistently monitored:

1) *Medical record reviews* are conducted on the "Road to Health" card issued to each child in South Africa by the Ministry of Health and which is required for accessing all services, including school and healthcare. We extract information from the card on routine health data including antenatal weight, height, blood pressure, HIV test results, CD4 count, rhesus factor results, test results for syphilis and other sexually transmitted infections, hemoglobin/anemia, tuberculosis, and preventative medications used, such as AZT and NVP. Delivery data, such as the date of delivery, baby weight and height, birth complications, and data from postnatal screening (vitamins dispensed, mother's TB and HIV test results, and the baby's weight, height, PCR test results, and immunizations) are also collected, as well as being documented on the Road to Health card.
2) *In-person interview assessments.* MAR are interviewed by trained research assistants after their first antenatal visit, 1 week after birth, and at 6 and 18 months. The baseline assessment takes between 60 and 90 min to complete, and covers family background, health, partnerships and sexual behaviors, disclosure, knowledge about HIV issues, daily routines, alcohol and drug use, and general knowledge about child care and infant feeding plans. All assessments are confidential, and are administered in Xhosa by a trained research assistant, supported by a computerized mobile phone interview (Tomlinson et al. 2009).

   Mobile phones are less expensive and less likely to be stolen than laptop computers, while also providing real-time data transfer as well as supervisory and project management information on the time and location of all assessment activities. This yields data collection with high integrity and facilitates calculation of the costs of data collection. The program improves data quality by performing simple logic and range validation as data are entered. Skip patterns are also automated, and sections of the survey can be automatically repeated the required number of times to cover, for example, the same questions about each of a woman's children or partners. Most important, the phones keep all data if no signal is available and automatically upload the data when a signal reappears. Secure Sockets Layering (SSL) is used to ensure that all data transferred between the device and the server are encrypted. Uploaded data are available for review, management and export via the web-based data platform. Only senior investigators are able to access the data platform. The servers hosting the data are located in Cape Town, South Africa, and offer full and redundant data protection and security.
3) *Monitoring the intervention dose and content.* Complementing paper records used to guide and monitor intervention delivery, a Mentor Mother's mobile phone records a time-location tag as she sends a message via the study mobile phone monitoring system whenever she is about to enter a household. A second time-location tag is created when the Mentor Mother leaves the household and sends a message noting the completion of the home visit. The mobile phone system then queries the Mentor Mother as to the content covered in the visit via brief questions on eight intervention messages and whether there was a crisis situation. Another mobile query assesses the degree to which the Mentor Mother believes that the mother was responsive to the message of the visit.

Measures

The intervention's effectiveness is currently being assessed over 18 months after a mother gives birth to her child. Outcomes are evaluated using routinely collected health data for mother and child, and on the basis of the four study assessments completed with the mother—one upon enrolment in the project, and three conducted postnatally (postpartum and 6-months and 18 months).

There is no single outcome that can definitively validate the success of the Philani + Mentor Mother program. For example, with only 23% of babies being HIV+ when ARV are not taken at childbirth, even shifting 100% of MAR to take 100% of her perinatal ARV appropriately would require thousands of MAR to demonstrate efficacy. We focus on the cumulative change on 14 indicators across 5 domains of maternal and child health caretaking, plus 10 additional indicators for the subset of mothers living with HIV. The primary outcome is a composite score that is calculated as the sum of indices for the presence (1) or absence (0) of maternal and child health and well-being across the five domains:

1) *Child health status* including birth weight within one standard deviation of WHO standards, normal development according to WHO norms, evidence of FAS at 18 months, and the Murray Global Rating of mother-infant interaction (Murray et al. 1996);
2) *Healthcare and monitoring* including the number of antenatal and postnatal clinic visits and additional care required if a mother is HIV-positive: monitoring and understanding CD4 count, adherence to all prescribed anti-retrovirals, and registration for her own healthcare twice annually;

3) *HIV-related preventive acts* including disclosure of HIV test results to partners, requesting partners to test, consistent use of condoms, and the following among HIV-positive mothers: Use of single feeding method until 6 months, baby testing at 6 weeks, baby prescribed antibiotics at 6 weeks, maternal AZT during pregnancy, NVP at birth, and AZT after birth, and disclosure of baby's status to partner or family;
4) *Mental health* including the Edinburgh Postnatal Depression Scale (Cox et al. 1987) and quality of life (QOL) assessed by the 12-item General Health Questionnaire (Goldberg et al. 1997); and
5) *Social support* including an adapted scale from Barrera and colleagues (1981) indicating instrumental, emotional, and childrearing support for healthy acts by family, neighbor, and community. The indices will be monitored throughout the intervention and over the 18-month follow-up period.

Data Analysis Plans

Intervention efficacy will be evaluated by comparing trajectories' composite outcome, as well as each individual indicator, over 18 months between the intervention and standard-of-care conditions. The composite scores will be compared using multilevel models, (i.e. hierarchical models or random effect models) which can account for clustering of repeated assessments within individuals and clustering of individuals within neighborhoods (Weiss 2005). Multilevel models can be applied to normally distributed and non-normally distributed outcomes; we will choose the most appropriate model based on the data. If data transformations sufficiently normalize the composite outcome, (e.g. a logarithmic transformation to reduce skewness), we will fit a linear model. If a normal assumption is not appropriate, we will categorize the data and use a longitudinal logistic regression for binary responses and longitudinal ordinal logistic regression to look for shifts in ordinal response levels between the intervention and control conditions. In all analyses, clustering within neighborhoods will be taken into account, and multiple imputation methods will be used for missing individual outcome scores (Schafer 1997).

While the composite score as primary outcome will provide a summary of the overall effectiveness of the intervention program, it is imperative that each outcome component also be examined, for more detailed interpretation of effectiveness. Therefore, each component outcome will be examined separately to determine whether, and in what direction, the intervention effected changes in each component.

Finally, dose–response analysis will also be conducted to test for relationships between the number of intervention sessions received, time of recruitment, and the composite score. The initial patterns of the outcome measures will be examined through descriptive summaries. If necessary, transformations will be conducted to approximate normality, homogeneous variance and linearity in the models.

Power Calculation

Power calculations were carried out in three steps. The first two steps adjusted for the three-level hierarchical design of the study: Repeated observations are nested within mothers and mothers are nested within neighborhoods. In addition to detecting intervention effects in the total sample, analyses were planned for each of the MAR subgroups; e.g., HIV-positive MAR. Therefore, the third step divided the calculated sample size by the fraction of women we expected to fall into the smallest MAR subgroup, approximately 30% of the MAR.

First, we calculated the necessary sample size per intervention arm (N*=156) to detect a standardized effect size of 0.40; i.e., a medium effect size (Cohen 1988), at 18 months with 80% power. Calculations were carried out in RMASS2 software (Hedeker et al. 1999) and assumed a Type I error of 0.05 for a two-sided test, three repeated measurements, 5% attrition between follow ups, and an autocorrelation (i.e., the correlation between adjacent repeated observations) of 0.50. Both the attrition rate and autocorrelation were estimated from our previous studies.

In the second step, the sample size per arm (N=177.5) was calculated as the product of N* and the variance inflation factor, $N = N^{*}x[1 + (m - 1)ICC]$, where m is the average number of women per neighborhood and the intracluster correlation (ICC) is estimated to be .01 from other behavioural studies (e.g., Todd et al. 2003).

Lastly, we calculate that 591.7 (= 177.5/0.30) MAR were needed per intervention arm (total sample size=1,184).

Results of Baseline Data Analysis

The neighborhood matching, randomization, and participant recruitment was successful in creating intervention and control neighborhoods and participant samples that were well-balanced on sociodemographic and background characteristics, as shown in Table 1. Mothers in the intervention and control neighborhoods were similar on most characteristics including *outcome-related measures*, such as rates of prior low-weight births (17%), visiting antenatal clinics (77%), HIV testing (92%), HIV-positive (26%), use of alcohol during pregnancy (26%, but 9% after recognizing their pregnancy), prenatal depression (17% clinically significant, 42% borderline), health quality of life (23% clinically significant), and social support measures; *matching criteria*, such as the type of housing (31% formal), source of water (53% on site), presence of flush toilet on premises (55%), the presence of electricity on the premises (90%), and

Table 1 Baseline characteristics of Philani Plus (+) study sample by intervention condition[(a)]

	Control (N=594)		Intervention (N=645)		Total (N=1,239)	
Mother's demographics						
Mean age, SD	26.3	5.6	26.5	5.5	26.4	5.5
Mean highest education level, SD	10.3	1.8	10.4	1.8	10.3	1.8
Marital status						
Single (n,%)	270	45.5	268	41.6	538	43.4
Married (n,%)	210	35.4	233	36.1	443	35.8
Not married but living together (n,%)	114	19.2	144	22.3	258	20.8
Employed (n,%)	104	17.5	130	20.2	234	18.9
Household income (N=1,195)						
Less than 2001 Rand (n,%)	301	51.9	334	54.3	635	53.1
2001 Rand or greater (n,%)	279	48.1	281	45.7	560	46.9
Living situation						
Formal housing vs. informal (n,%)	191	32.2	198	30.7	389	31.4
Water on site vs. not (n,%)	327	55.1	334	51.8	661	53.3
Flush toilet vs. other types (n,%)	343	57.7	341	52.9	684	55.2
Have electricity (n,%)	543	91.4	570	88.4	1113	89.8
Median days hungry, range						
Mother	1	0–7	0	0–7	0	0–7
Children in household	0	0–7	0	0–7	0	0–7
Mother's health						
Booked appointment at antenatal clinic (N=1,145)[(b)] (n,%)	376	75.2	505	78.3	881	76.9
Median # weeks mother should return to clinic post-birth, range (N=788)[(b)]	3	1–10	3	1–8	3	1–10
Chronic illness rates						
Diabetes (n,%)	14	2.4	14	2.2	28	2.3
Hypertension (n,%)	61	10.3	62	9.6	123	9.9
Disability (N=1,145)[(b)] (n,%)	4	0.8	10	1.6	14	1.2
Asthma (N=1,145)[(b)] (n,%)	19	3.8	34	5.3	53	4.6
At least one (N=1,145)[(b)] (n,%)	108	21.6	158	24.5	266	23.2
Mean count of chronic illnesses, range (n=1,145)[(b)]	0.2	0–4	0.2	0–4	0.2	0–4
TB in lifetime						
Never tested (n,%)	384	64.6	439	68.2	823	66.5
Negative (n,%)	160	26.9	154	23.9	314	25.4
Positive (n,%)	50	8.4	51	7.9	101	8.2
Treated, if positive (N=101) (n,%)	50	100	49	96.1	99	98.0
TB during pregnancy						
Not tested (n,%)	475	80	509	79.4	984	79.7
Negative (n,%)	118	19.9	128	20	246	19.9
Positive (n,%)	1	0.2	4	0.6	5	0.4
Treated for TB, this pregnancy (N=5) (n,%)	1	100	3	75	4	80
Taking/took during pregnancy						
Multivitamin (n,%)	265	44.6	267	41.4	532	42.9
Folic acid (n,%)	330	55.6	367	56.9	697	56.3
Iron tonic/tablets (n,%)	284	47.8	307	47.6	591	47.7
Calcium (n,%)	214	36	241	37.4	455	36.7
None of these (n,%)	149	25.1	160	24.8	309	24.9
Previous LBW babies (N=768 previous birth) (n,%)	69	18.4	61	15.5	130	16.9
Traditional medicine (N=826 responded)[(b)] (n,%)	161	44.8	229	49	390	47.2
Substance use						
Drank any alcohol during pregnancy (N=1,214 responded) (n,%)	153	26.9	172	26.7	325	26.8

Table 1 (continued)

	Control (N=594)		Intervention (N=645)		Total (N=1,239)	
Tobacco (n,%)	17	2.9	25	3.9	42	3.4
Cannabis (N=1,145)[(b)] (n,%)	0	0	1	0.2	1	0.1
HIV, disclosure & partners						
Tested for HIV (n,%)	550	92.6	591	91.6	1141	92.1
Positive result (N=1,132 received result) (n,%)	146	26.7	149	25.5	295	26.1
Plan to tell clinic sister HIV status at birth (N=271)[(b)] (n,%)	123	100	148	100	271	100
Mean # people disclosed to, range (N=272)[(b)]	4.2	0–57	3.8	0–30	4	0–57
If HIV positive, told partner (N=245)[(b)] (n,%)	91	82	99	73.9	190	77.6
Current sexual partner(s) (N=1,145)[(b)] (n,%)	469	94	596	92.5	1065	93.2
Had HIV+sex partner past 3 months (N=835)[(b)] (n,%)	41	11.4	46	9.7	87	10.4
Asked sex partner to test for HIV (N=834)[(b)] (n,%)	295	82.2	391	82.3	686	82.3
Infant feeding						
Feeding method(s) plan to use 1st 6 mo. (N=1,145)[(b)]						
Only breast feeding (n,%)	304	61	421	65.9	725	63.8
Only formula feeding (n,%)	141	28.3	172	26.9	313	27.5
Breast feed & formula (mixed) (n,%)	53	10.6	46	7.2	99	8.7
Mental health						
Health quality of life (GHQ; higher=worse, possible range=0–12) (N=867)[(b)]						
Median score, range	0	0–12	0	0–12	0	0–12
Caseness (score>6) (n,%)	86	22.8	109	22.3	195	22.5
Prenatal depression (EPDS; higher=worse, possible range=0–30) (N=1,145)[(b)]						
Median score, range	10	0–30	10	0–28	10	0–30
Significant depression (score>18) (n,%)	81	16.2	109	16.9	190	16.6
Possible depression (score>12) (n,%)	200	40	275	42.6	475	41.5
Social support						
Median number of close friends/relatives, range	2	0–10	2	0–10	2	0–10
Median past month frequency contact, range	9.5	0–60	10	0–60	10	0–60
Median times received support past week, range	2	0–30	2	0–30	2	0–30

No significant differences exist between control and intervention conditions.

[(a)] Table includes measures asked at Baseline assessment for regular-entry participants (REP) and a subset of those measures asked retrospectively for late-entry participants (LEP).

[(b)] Measures assessed for REP only: Control (N=500), Intervention (N=645), Total (N=1,145)

length of residence in Cape Town; *demographics*, including age (about 26), marital status (36% married and living with partner), education, employed (19%), and household income (53% earn less than 2001 Rand per month, approximately 294 $USD); and *general health*, including rates of HIV infection (26%), and chronic illnesses (23% had at least one of four assessed).

Discussion

The neighborhood recruitment for this community-level intervention trial has been successful in identifying a large sample of mothers at risk in South African townships using recruiters drawn from the neighborhoods. However, recruitment was more rapid in the intervention neighborhoods where the Mentor Mother CHWs were instrumental in helping to identify potential participants by building trust and relationships within the communities and by providing an additional "sweep" of neigborhood households when intervening with participants recruited earlier in the study. Although this presents a limitation to research methods, it does demonstrate the potential for Mentor Mother CHW home visitors to effectively identify, engage, and deliver interventions to mothers at risk for multiple health risks.

The results of baseline data analysis also demonstrate that multiple health risks are present at the community level, although not necessarily manifesting as a high prevalence of comorbidity at the individual level. However,

the Mentor Mother CHW's success in engaging and recruiting all mothers in a community into the intervention is supported by the CHW's ability to address the multiple health risks and challenges present within a community that mothers and their children may experience, regardless of co-occurrence within any individual family.

Another innovative aspect of this study is the use of inexpensive and widely available mobile handsets, mobile survey software and existing cellular networks to provide an electronic data collection platform (Tomlinson et al. 2009). The value of this inexpensive innovation is enhanced given the low resource setting in which the research and intervention is being conducted. Many of the study clinics do not have desktop computers, landline telephones or even easy vehicle access. Yet, through the mobile platform, many of the established benefits of electronic data collection are made available despite the challenging circumstances. The same principles and tools used for mobile data collection are also applied to intervention delivery support and fidelity monitoring for the CHWs and their supervisory teams. These strategies demonstrate the potential for mobile phones to support the ongoing delivery, monitoring, and evaluation of interventions by CHWs and others, which are often considered to be too burdensome and costly to implement routinely. We anticipate that this system will improve the sustained efficacy of the intervention and could be scaled up to support larger-scale effectiveness trials. This study is not designed to test the impact of the mobile phone intervention monitoring and support system but another trial is planned that would specifically assess this compared to a non-phone comparison group of CHWs.

Conclusion

Donor funding has allowed broad diffusion of HIV prevention, treatment, and care. Yet, developing countries cannot broadly sustain categorically funded programs, without addressing concurrent and intersecting health challenges, such as alcohol abuse, poor mental health, and malnutrition. The healthcare budgets in the developing world are so low as to require horizontally integrated care. Yet, efficacious HIV prevention programs have been designed for vertically integrated healthcare systems only (Global Issues 2010). HIV prevention programs typically address a single outcome (reducing HIV transmission) and are categorically funded and vertically integrated at the clinic, hospital, township, provincial, and national levels. The Global Fund, the U.S. HIV funding (PEPFAR), World Bank, and many private donors fund HIV prevention and care only. In 33 of 41 African countries (70%), the total annual health care budget is less than $30 USD per person and only two countries spend more than 10% of their annual budgets on health care (Ooms 2006). Although South Africa spends $390 USD/person annually (8.6% of budget), it is insufficient to fund HIV categorically, when there is a 30% infection rate among pregnant women. Concurrently, more than 60% of the children who die under the age of 5 years (12% of children), die from malnutrition, dehydration, other infections, or complications related to alcohol use (Department of Health, Pretoria 2003). Prevention must be integrated for the major health risks in local communities.

Preventive services offered by paraprofessional CHWs can supplement an overwhelmed health care system that cannot meet Africa's HIV, alcohol, and nutritional needs and certainly cannot meet the needs of the range of diseases confronting developing nations. The health care system has the responsibility for HIV care, but the system is overwhelmed by HIV: more than 1 million health care workers are needed immediately (Global Issues 2010). It will cost billions of dollars to train the health care providers needed. Yet, 65,000 physicians and 75,000 nurses immigrated to the UK during the 1990s and the drain continues (Rotheram-Borus et al. 2009). Health care alone cannot meet the challenge of HIV.

From the consumer's perspective, the health care provider is not a prime resource for prevention. This is certainly true of the one billion people who do not have access to health care globally. Historically, and certainly in the context of overwhelming and overlapping HIV and TB epidemics, systems for delivering health care are not consumer-friendly. Clinics have long waiting lines and limited resources, are difficult to access, and, in many countries, are expensive (Rotheram-Borus et al. 2009). Consumers often struggle to know whether a problem is severe enough to require treatment; the responsibility and choice for seeking care is with the consumer. Consumers seeking care often face a complex process that involves getting referrals to find the right clinic, dealing with long waiting times for and transportation to appointments, taking time off from work or taking children out of school to accommodate the health care provider's available appointment times, paying high rates that are frequently not covered by insurance, and wondering how long the health appointments will last and whether it will successfully address the problem about which they are concerned. These are difficult challenges to overcome. The paraprofessional CHW Mentor Mother is one strategy to confront these challenges.

The U.S. has recently authorized $1.5 billion in the health care reform bill for the Nurse Home Visiting Partnerships (America's Affordable Health Choices Act of 2009). If successful, the Philani program offers a more cost-effective strategy for addressing maternal and child health

issues, especially in low-resource settings. By placing Mentor Mothers in geographically defined areas, the social capital of the neighborhood is built over time as the parenting skills of the mothers are supported. This contributes to the long-term development of the community and the country in a sustainable way.

References

America's Affordable Health Choices Act of 2009. H.R. 3200 Library of Congress.

Bang, A. T., Reddy, H. M., Deshmukh, M. D., Baitule, S. B., & Bang, R. A. (2005). Neonatal and infant mortality in the ten years (1993 to 2003) of the Gadchiroli field trial: Effect of home-based neonatal care. *Journal of Perinatology, 25*, S92–S107.

Barbarin, O. A. (2003). Social risks and child development in South Africa: A nation's program to protect the human rights of children. *American Journal of Orthopsychiatry, 73*, 248–259.

Barrera, M., Sandler, I., & Ramsay, T. (1981). Preliminary development of a scale of social support: Studies on college students. *American Journal of Community Psychology, 9*, 435–447.

Berggren, G., Alvarez, M., Genece, E., Amadee-Gedeon, P., & Henry, M. (1984). *The nutrition demonstration foyer: A model for combating malnutrition in Haiti. Hoviprep Monostraph Series #2, International Food and Nutrition Program of MIT.* Boston: MIT.

Bloch, M., Althabe, F., Onyamboko, M., Kaseba-Sata, C., Castilla, E. E., Freire, S., et al. (2008). Tobacco use and secondhand smoke exposure during pregnancy: An investigative survey of women in 9 developing nations. *American Journal of Public Health, 98*, 1833–1840. doi:10.2105/AJPH.2007.117887.

Bryant, K. J. (2006). Expanding research on the role of alcohol consumption and related risks in the prevention and treatment of HIV/AIDS. *Substance Use & Misuse, 41*, 1465–1507. doi:10.1080/10826080600846250.

Chaguturu, S., & Vallabhaneni, S. (2005). Aiding and abetting–nursing crises at home and abroad. *New England Journal of Medicine, 353*, 1761–1763. doi:10.1056/NEJMp058201.

City of Cape Town official website: 2001 Census. 2010 [http://www.capetown.gov.za/en/stats/2001census/Pages/default.aspx]

City of Cape Town official website: Economic statistics. (2011). [http://www.capetown.gov.za/en/ehd/Pages/EconomicStatistics.aspx]

City of Cape Town official website: Local government services. (2011). [http://www.capetown.gov.za/en/Services/Pages/default.aspx]

Cohen, J. (1988). *Statistical power analysis for the behavioral sciences*. Mahwah, NJ: Lawrence Erlbaum.

Cooper, P. J., Tomlinson, M., Swartz, L., Woolgar, M., Murray, L., & Molteno, C. (1999). Post-partum depression and the mother-infant relationship in a South African peri-urban settlement. *The British Journal of Psychiatry, 175*, 554–558.

Cox, J. L., Holden, J. M., & Sagovsky, R. (1987). Detection of postnatal depression: Development of the 10-item Edinburgh Postnatal Depression Scale. *The British Journal of Psychiatry, 150*, 782–786.

Dawson, D., Grant, B., & Stinson, F. (2005). The AUDIT-C: screening for alcohol use disorders and risk drinking in the presence of other psychiatric disorders. *Comprehensive Psychiatry*, 405–416. doi: 10.1016/j.comppsych.2005.01.006

Department of Health, Pretoria, South Africa: South Africa Demographic and Health Survey 2003, Preliminary Report [http://www.doh.gov.za/facts/sadhs2003/part1.pdf]

Dermen, K. H., & Cooper, M. L. (2000). Inhibition conflict and alcohol expectancy as moderators of alcohol's relationship to condom use. *Experimental and Clinical Psychopharmacology, 8*, 198–206.

Durantini, M. R., Albarracín, D., Mitchell, A. L., Earl, A. N., & Gillette, J. C. (2006). Conceptualizing the influence of social agents of behavior change: A meta-analysis of the effectiveness of HIV-prevention interventionists for different groups. *Psychological Bulletin, 132*, 212–248. doi:10.1037/0033-2909.132.2.212.

Frontline, (2007). [http://www.frontline.org.za/articles/alcohol_abuse%20in%20SA.htm]

Global issues – Social, political, economic and environmental issues that affect us all: Health issues 2010 [http://www.globalissues.org/issue/587/health-issues]

Goldberg, D. P., Gater, R., Sartorius, N., Ustun, T. B., Piccinelli, M., Gureje, O., et al. (1997). The validity of two versions of the GHQ in the WHO study of mental illness in general health care. *Psychological Medicine, 27*, 191–197.

Gomby, D. S., Culross, P. L., & Behrman, R. E. (1999). Home visiting: Recent program evaluations–analysis and recommendations. *The Future of Children, 9*, 4–26, 195–223.

Groenewald, P., Vos, T., Norman, R., Laubscher, R., van Walbeek, C., Saloojee, Y., et al. (2007). Estimating the burden of disease attributable to smoking in South Africa in 2000. *South African Medical Journal, 97*, 674–681.

Haines, A., Sanders, D., Lehmann, U., Rowe, A. K., Lawn, J. E., Jan, S., et al. (2007). Achieving child survival goals: Potential contribution of community health workers. *The Lancet, 369*, 2121–2131.

Hall, L. A., Sachs, B., & Rayens, M. K. (1998). Mothers' potential for child abuse: The roles of childhood abuse and social resources. *Nursing Research, 47*, 87–95.

Hedeker, D., Gibbons, R., & Waternaux, C. (1999). Sample size estimation for longitudinal designs with attrition: Comparing time-related contrasts between two groups. *Journal of Educational and Behavioral Statistics, 24*, 70–93. doi:10.3102/10769986024001070.

Kelly, R. H., Russo, J., Holt, V. L., Danielsen, B. H., Zatzick, D. F., Walker, E., et al. (2002). Psychiatric and substance use disorders as risk factors for low birth weight and preterm delivery. *Obstetrics & Gynecology, 100*, 297–304.

Kodituwakku, P. W., Kalberg, W., & May, P. A. (2001). The effects of prenatal alcohol exposure on executive functioning. *Alcohol Research & Health, 25*, 192–199.

le Roux, I., le Roux, K., Comulada, W. S., Greco, E., Desmond, K., Mbewu, N., et al. (2010). Home visits by neighborhood Mentor Mothers provide timely recovery from childhood malnutrition in South Africa: Results from a randomized controlled trial. *Nutrition Journal, 9*, 56.

le Roux, I., le Roux, K., Mbeutu, K., Comulada, W. S., Desmond, K. A., & Rotheram-Borus, M. J. (2011). A randomized controlled trial of home visits by neighborhood mentor mothers to improve children's nutrition in South Africa. *Vulnerable Children and Youth Studies, 6*, 91–102.

Lewin, S., Lavis, J. N., Oxman, A. D., Bastías, G., Chopra, M., Ciapponi, A., et al. (2008). Supporting the delivery of cost-effective interventions in primary health-care systems in low-income and middle-income countries: An overview of systematic reviews. *The Lancet, 372*, 928–939.

Manandhar, D. S., Osrin, D., Shrestha, B. P., Mesko, N., Morrison, J., Tumbahangphe, K. M., et al. (2004). Effect of a participatory intervention with women's groups on birth outcomes in Nepal: Cluster-randomised controlled trial. *The Lancet, 364*, 970–979.

Marsh, D. R., Schroeder, D. G., Dearden, K. A., Sternin, J., & Sternin, M. (2004). The power of positive deviance. *British Medical Journal, 329*(7475), 1177–1179. doi:10.1136/bmj.329.7475.1177.

May, P. A., Brooke, L., Gossage, J. P., Croxford, J., Adnams, C., Jones, K. L., et al. (2000). Epidemiology of fetal alcohol syndrome in a South African community in the Western Cape province. *American Journal of Public Health, 90*, 1905–1912.

May, P. A., Gossage, J. P., White Country, M., Goodhart, K., Decoteau, S., Trujillo, P. M., et al. (2004). Alcohol consumption and other maternal risk factors for fetal alcohol syndrome among three distinct samples of women before, during, and after pregnancy: The risk is relative. *American Journal of Medical Genetics, 127C*, 10–20.

May, P. A., Gossage, J. P., Brooke, L. E., Snell, C. L., Marais, A. S., Hendricks, L. S., et al. (2005). Maternal risk factors for fetal alcohol syndrome in the Western Cape province of South Africa: A population-based study. *American Journal of Public Health, 95*, 1190–1199. doi:10.2105/AJPH.2003.037093.

Mermin, J., Lule, J., Ekwaru, J. P., Malamba, S., Dowing, R., Ransom, R., et al. (2004). Effect of cotrimoxazole prophylaxis on morbidity, mortality, cd4-cell count, and viral load in HIV infection in rural Uganda. *The Lancet, 364*, 1428–1434.

Morojele, N., Kachieng'a, M., Mokoko, E., Nkoko, M., Parry, C., Nkowane, A., et al. (2006). Alcohol use and sexual behaviour among risky drinkers and bar and shebeen patrons in Gauteng province, South Africa. *Social Science & Medicine, 62*, 217–227.

Morrison, J., Tamang, S., Mesko, N., Osrin, D., Shrestha, B., Manandhar, M., et al. (2005). Women's health groups to improve perinatal care in rural Nepal. *BMC Pregnancy and Childbirth, 5*, 6.

Murray, L., Fiori-Cowley, A., Hooper, R., & Cooper, P. (1996). The impact of postnatal depression and associated adversity on early mother-infant interactions and later infant outcome. *Child Development, 67*, 2512–2526.

Myer, L., Ehrlich, R. I., & Susser, E. S. (2004). Social epidemiology in South Africa. *Epidemiologic Reviews, 26*, 112–123. doi:10.1093/epirev/mxh004.

O'Connor, M. J., & Kasari, C. (2000). Prenatal alcohol exposure and depressive features in children. *Alcoholism: Clinical and Experimental Research, 24*, 1084–1092. doi:10.1111/j.1530-0277.2000.tb04654.x.

O'Connor, M. J., & Whaley, S. E. (2007). Brief intervention for alcohol use by pregnant women. *American Journal of Public Health, 97*, 252.

O'Connor, M. J., Shah, B., Whaley, S., Cronin, P., Gunderson, B., & Graham, J. (2002). Psychiatric illness in a clinical sample of children with prenatal alcohol exposure. *American Journal of Drug & Alcohol Abuse, 28*, 743–754.

Olds, D. L., Robinson, J., O'Brien, R., Luckey, D. W., Pettitt, L. M., Henderson, C. R., Jr., et al. (2002). Home visiting by paraprofessionals and by nurses: A randomized controlled trial. *Pediatrics, 110*, 486–496. doi:10.1542/peds.110.3.486.

Olds, D. L., Sadler, L., & Kitzman, H. (2007). Programs for parents of infants and toddlers: Recent evidence from randomized trials. *Journal of Child Psychology and Psychiatry, 48*, 355–391. doi:10.1111/j.1469-7610.2006.01702.x.

Ooms, G. (2006). Health development versus medical relief: The illusion versus the irrelevance of sustainability. *PLoS Medicine, 3*, e345. doi:10.1371/journal.pmed.0030345.

Riley, E. P., & McGee, C. L. (2005). Fetal alcohol spectrum disorders: An overview with emphasis on changes in brain and behavior. *Experimental Biology and Medicine (Maywood), 230*, 357–365.

Rollins, N. C., Coovadia, H. M., Bland, R. M., Coutsoudis, A., Bennish, M. L., Patel, D., et al. (2007). Pregnancy outcomes in HIV-infected and uninfected women in rural and urban South Africa. *Journal of Acquired Immune Deficiency Syndromes, 44*, 321.

Rotheram-Borus, M. J., & Duan, N. (2003). Next generation of preventive interventions. *Journal of the American Academy of Child & Adolescent Psychiatry, 42*, 518–526. doi:10.1097/01.CHI.0000046836.90931.E9.

Rotheram-Borus, M. J., Lee, M., Lin, Y., & Lester, P. (2004a). Six-year intervention outcomes for adolescent children of parents with the human immunodeficiency virus. *Archives of Pediatrics and Adolescent Medicine, 158*, 742–748.

Rotheram-Borus, M. J., Swendeman, D., Comulada, W. S., Weiss, R. E., Lee, M., & Lightfoot, M. (2004b). Prevention for substance-using HIV-positive young people: Telephone and in-person delivery. *Journal of Acquired Immune Deficiency Syndromes, 37*, S68–S77.

Rotheram-Borus, M. J., Flannery, D., Rice, E., & Lester, P. (2005). Families living with HIV. *AIDS Care, 17*, 978–987. doi:10.1080/09540120500101690.

Rotheram-Borus, M. J., Swendeman, D., Flannery, D., Rice, E., Adamson, D. M., & Ingram, B. (2009). Common factors in effective HIV prevention programs. *AIDS and Behavior, 13*, 399–408.

Sazawal, S., & Black, R. E. (2003). Effect of pneumonia case management on mortality in neonates, infants, and preschool children: A meta-analysis of community-based trials. *The Lancet Infectious Diseases, 3*, 547–556.

Schafer, J. L. (1997). *Analysis of incomplete multivariate data*. New York: Chapman & Hall.

Sood, B., Delaney-Black, V., Covington, C., Nordstrom-Klee, B., Ager, J., Templin, T., et al. (2001). Prenatal alcohol exposure and childhood behavior at age 6 to 7 years: I. dose-response effect. *Pediatrics, 108*, E34.

South Africa National Department of Health, Policy and Guidelines for the Implementation of the PMTCT Programme 2008. [http://www.doh.gov.za/docs/policy/pmtct.pdf]

Sternin, M., Sternin, J., & Marsh, D. (1998). Designing a community-based nutrition program using the Hearth model and the positive deviance approach – a field guide. *Save the Children.* [http://www.positivedeviance.org/pdf/fieldguide.pdf]

Sweet, M. A., & Appelbaum, M. I. (2004). Is home visiting an effective strategy? A meta-analytic review of home visiting programs for families with young children. *Child Development, 75*, 1435–1456. doi:10.1111/j.1467-8624.2004.00750.x.

Teasdale, C., & Besser, M. (2008). Enhancing PMTCT programmes through psychosocial support and empowerment of women: The mothers2mothers model of care. *Southern African Journal of HIV Medicine, 9*, 60–64.

Todd, J., Carpenter, L., Li, X., Nakiyingi, J., Gray, R., & Hayes, R. (2003). The effects of alternative study designs on the power of community randomized trials: Evidence from three studies of human immunodeficiency virus prevention in East Africa. *International Journal of Epidemiology, 32*, 755–762.

Tomlinson, M. (2010). Family-centered HIV interventions: Lessons from the field of parental depression. *Journal of the International AIDS Society, 13*, S9.

Tomlinson, M., Solomon, W., Singh, Y., Doherty, T., Chopra, M., Ijumba, P., et al. (2009). The use of mobile phones as a data collection tool: A report from a household survey in South Africa. *BMC Medical Informatics and Decision Making, 9*, 51.

UNAIDS 2007 AIDS Epidemic Update [http://data.unaids.org/pub/EPISlides/2007/2007_epiupdate_en.pdf]

Verplanken, B., & Wood, W. (2006). Interventions to break and create consumer habits. *Journal of Public Policy & Marketing, 25*, 90–103.

Walley, J., Lawn, J. E., Tinker, A., de Francisco, A., Chopra, M., Rudan, I., et al. (2008). Primary health care: Making Alma-Ata a reality. *The Lancet, 372*, 1001–1007. doi:10.1016/S0140-6736(08)61409-9.

Weiss, R. (2005). *Modeling longitudinal data*. New York: Springer Verlag.

Were, W., Mermin, J., Bunnel, R., Elwaru, J. P., & Kaharuza, F. (2003). Homebased model for HIV voluntary counseling and testing. [Letter to the editor.]. *The Lancet, 361*, 1569.

World Health Organization. (2008). *Task shifting: Global recommendations and guidelines.* Retrieved August 10, 2008, from UNAIDS Web site: http://data.unaids.org/pub/Manual/2007/ttr_taskshifting_en.pdf.

World Health Organization & UNICEF, (2009). Co-trimaxazole prophylaxis for HIV-exposed and HIV-infected infants and children: Practical approaches to implementation and scale up. [http://www.unicef.org/aids/files/CotrimoxazoleGuide_2009.pdf]

World Health Organization. Division of Family and Reproductive Health. Integrated Management of Childhood Illness. 2010 [http://www.afro.who.int/imci/index.html]

Zere, E., & McIntyre, D. (2003). Inequities in under-five child malnutrition in South Africa. *International Journal for Equity in Health, 2*, 7. doi:10.1186/1475-9276-2-7.
